# Supplementary material for: A stochastic mechanism drives fast substrate translocation in the AAA+ machine ClpB
Source: Nat Commun. 2026 Jan 21;17:1773. doi: 10.1038/s41467-026-68478-1 (PMC12917000; doi:10.1038/s41467-026-68478-1)
Supplement: Supplementary file 1 — Supplementary Information [file 41467_2026_68478_MOESM1_ESM.pdf]

Supplementary Information for

A Stochastic Mechanism Drives Fast Substrate Translocation in the AAA+ Machine ClpB

Remi Casier[1], Dorit Levy[1], Inbal Riven[1], Yoav Barak[2], Gilad Haran[1]*

[1]Department of Chemical and Biological Physics, Weizmann Institute of Science, Rehovot 761001, Israel

[2]Chemical Research Support, Weizmann Institute of Science, Rehovot, 761001, Israel

* to whom correspondence should be addressed.

Table of Contents

Supplementary Methods 5

Liposome permeability. 5

Calculation of the expected FRET efficiencies 5

Calculation of the expected number of proteins inside a liposome. 6

Fluorescence anisotropy decay experiments. 7

Distribution of labeled casein inside liposomes 8

Derivation of leak and background corrections 8

Leak factor determination 9

Determining inter-acceptor energy transfer 10

Fluorescence correlation spectroscopy 10

Supplementary Figures 12

Supplementary Figure 1. Extrusion produces monodisperse large unilamellar vesicles 12

Supplementary Figure 2. ATPγS increases the apparent affinity of casein to ClpB 13

Supplementary Figure 3. ClpB and κ-casein freely diffuse inside immobilized liposomes 14

Supplementary Figure 4. ATP readily diffuses in and out of DMPC liposomes 15

Supplementary Figure 5. ClpB remains active after point mutations and fluorescence labeling 16

Supplementary Figure 6. Photobleaching experiments on CF660R labeled casein demonstrate casein overwhelmingly contains at most a single acceptor 17

Supplementary Figure 7. The acceptor remains fluorescent throughout the trajectory 18

Supplementary Figure 8. Calculated FRET efficiencies for NBD2 labeled ClpB demonstrate that high FRET efficiency events can only occur if casein passes through the NBD2 ring 19

Supplementary Figure 9. Sample trajectories of translocation of casein through NBD1 labeled ClpB in the presence of 2 mM ATP 20

Supplementary Figure 10. Sample trajectories of translocation of casein through NBD2 labeled ClpB in the presence of 2 mM ATP 21

Supplementary Figure 11. Sample trajectories of translocation of casein through NBD1 labeled ClpB in the presence of 2 mM ATPγS 22

Supplementary Figure 12. Sample trajectories of translocation of casein through NBD2 labeled ClpB in the presence of 2 mM ATPγS 23

Supplementary Figure 13. Sample trajectories of translocation of casein as a function of temperature 24

Supplementary Figure 14: Event dwell time is weakly affected by temperature 25

Supplementary Figure 15. Sample trajectories of translocation of casein as a function of ATP concentration 26

Supplementary Figure 16. Event dwell time is weakly affected by ATP concentration..........27
Supplementary Figure 17. Event frequency directly correlates with ATP concentration.........28
Supplementary Figure 18: Acceptor photobleaching study on double-acceptor labeled ClpB demonstrates negligible inter-acceptor energy transfer..29
Supplementary Figure 19. Sample trajectories of Cy3B-labeled casein translocating through a double-labeled ClpB...30
Supplementary Figure 20: Characterization of the different types of interactions in the presence of ATP...31
Supplementary Figure 21. Cumulative dwell time histogram of all combined events from the three-color experiments..31
Supplementary Figure 22. Samples of complete forward translocation type I events.............32
Supplementary Figure 23. Samples of complete reverse translocation type II events.............33
Supplementary Figure 24. Samples of partial translocation types III (top) and IV (bottom) events 34
Supplementary Figure 25. Samples of binding types V (top) and VI (bottom) events............35
Supplementary Figure 26: Characterization of the different types of events in the three-color experiments in the presence of ATPγS..36
Supplementary Figure 27: Calibration of the temperature cell..37
Supplementary Figure 28. SOPC vesicles are permeable to ATP at 10 °C............................38
Supplementary Figure 29. 15:0 PC vesicles are permeable to ATP at 32 °C..........................38
Supplementary Figure 30: Global analysis of the time-resolved fluorescence anisotropy decay curves of ClpB 359..39
Supplementary Figure 31. The per-molecule Cy3B average leak factor into the acceptor channel in two-color FRET experiments..40
Supplementary Figure 32. The per-molecule average leak factors in the three-color FRET experiments...40
Supplementary Tables...41
Supplementary Table 1. Summary of the number of labeled ClpB molecules sampled under various experimental conditions...41
Supplementary Table 2. Dwell time characterization as a function of temperature and ATP concentration..41
Supplementary Table 3. Parameters retrieved from the triexponential fits of the combined (NBD1 and NBD2 labeled ClpB) two-color dwell time CDF...42
Supplementary Table 4. Parameters retrieved from the triexponential fits of the two-color dwell time CDF as a function of ATP concentration...42
Supplementary Table 5. Parameters retrieved from the exponential fits of the frequency of events as a function of ATP concentration..42
Supplementary Table 6. Summary of parameters characterizing the different types of events.43

Supplementary Table 7. Parameters retrieved from the triexponential fit of the three-color dwell time CDF in the presence of ATP and ATPγS.....43
Supplementary Table 8. Translocation velocities estimated from pore loop dynamics.....43
Supplementary Table 9. Size distribution of extruded liposomes.....44
Supplementary Table 10. Summary of the steady-state fluorescence anisotropy of fluorescently labeled ClpB.....44
Supplementary Table 11. Parameters retrieved from the global analysis of the fluorescence anisotropy decays of Cy3B-labeled ClpB.....44
Supplementary Table 12: Parameters retrieved from the FCS curves of Cy3B at different temperatures.....45
Supplementary Table 13. ATPase activity of ClpB in the presence of 2 mM ATP.....45
Supplementary Table 14. Parameters retrieved from the fit of the ATPase activity by the Hill equation.....45
Supplementary References.....46

Supplementary Methods

Liposome permeability. Liposomes constituting lipids near their liquid-to-gel transition temperature are known to remain permeable to small molecules, such as nucleotides, while retaining larger molecules, such as DNA and proteins (2). The permeability of DMPC liposomes to ATP at the phase transition temperature of 22.5 °C was verified using surface-immobilized liposomes containing a fluorescently labeled adenylate kinase variant (AK 82V, mutations: 82V, 73C, 142C, dyes: AF488, AF594) inside a flow cell. AK 82V displays a large conformational shift from a primarily low FRET efficiency state, in the absence of ATP, to a high FRET efficiency state upon the addition of ATP (3), making it an ideal sensor for the presence of ATP. After encapsulation and immobilization without ATP (50 mM TRIS, 100 mM KCl, 15 mM $MgCl_2$, pH 8), trajectories of liposomes containing a double-labeled AK were monitored until photobleaching occurred using a circularly polarized 485 nm laser (ca. 200 nW). The histogram of apparent FRET efficiency values revealed a major population of AK in the low FRET efficiency state (E ~ 0.4, Supplementary Figure 4a). The flow cell was then flushed with a buffer solution containing 2 mM ATP, resulting in a shift in the FRET efficiency to 0.68 (Supplementary Figure 4b) and demonstrating that ATP readily penetrated the liposomes. The reversibility of the process was then demonstrated by flushing with a buffer solution without ATP (Supplementary Figure 4c), resulting in a shift back to low FRET efficiency. This process could then be repeated with the same outcome (Supplementary Figure 4d).

Calculation of the expected FRET efficiencies. The expected FRET efficiencies ($\varepsilon_{\mathrm{calc}}$) were calculated as a function of translocation depth through the lumen of ClpB using a hexameric model of the protein based on PDB 1QVR (4). The translocation depth was measured as the distance from the 'top' of the NTD (i.e., at a depth of 0 nm) to the 'bottom' of the NBD2, corresponding to a depth of 12.63 nm. $\varepsilon_{\mathrm{calc}}$ values were determined for the line passing directly through the center of the lumen, as well as the limiting values based on the boundaries of the pore. The distance d separating the reference labeling location on ClpB and the interior of the lumen was measured in the following manner. First, the structure of ClpB was oriented such that the z-axis passed directly through the center of the lumen. Next, the structure of ClpB was divided into 150 even slices orthogonal to the z-axis. For each slice, the distances from the reference labeling location on ClpB to the center of the lumen and the atoms lining the interior of the pore were determined. The distances corresponding to the atoms closest and furthest from the reference point in each slice were taken as the limiting boundaries of the pore. $\varepsilon_{\mathrm{calc}}$ values were determined by inputting the measured distances into

$$\varepsilon_{calc}(two-color)=\frac{1}{1+\left(\frac{d}{R_0}\right)^6},$$

where R_0 is the Förster radius provided in the main text. The total $\varepsilon_{\mathrm{calc}}$ value in the three-color map was determined as

$$\varepsilon_{calc}(three-color) = \frac{1}{1+\dfrac{1}{\left(\dfrac{R_{0,1}}{d_1}\right)^6+\left(\dfrac{R_{0,2}}{d_2}\right)^6}},$$

where the subscripts 1 and 2 refer to the two acceptors (5).

Calculation of the expected number of proteins inside a liposome. Liposomes were prepared by the reconstitution of dried lipids using a solution containing a mixture of ClpB and casein. Assuming the proteins are randomly distributed throughout the solution, the number of proteins contained inside an individual liposome obeys a Poisson distribution ($P(i,\lambda) = \lambda^i e^{-\lambda}/i!$), where i is the number of proteins in a given liposome and λ is the average number of proteins contained per liposome. The expectation value, λ, is readily calculated using the concentration of proteins in solution (c_P) and the volume of solution contained within a liposome (V_L), with the relation $\lambda = c_P \times V_L$. The diameter of the liposomes (= 119 nm, see Supplementary Figure 1) was used to estimate the interior volume of the liposomes. Assuming a spherical shape, the volume was calculated using the radius of the liposome minus the thickness of the lipid bilayer (= 3.7 nm at 30 °C for DMPC (6)). An example calculation for the expected number of caseins per liposome, prepared from an initial solution of 3 μM casein, is provided below.

$$\lambda = c_P V_L = (3\ \mu M) \times \left(\frac{4}{3}\pi\left(\frac{119nm}{2} - 3.7nm\right)^3\right)$$

$$= 3\ \mu M \left(\frac{\frac{10^{-6}mol}{L}}{1\ \mu M}\right)\left(\frac{1L}{10^{-3}m^3}\right)\left(\frac{1m}{10^9 nm}\right)^3\left(\frac{6.022\times10^{23}proteins}{1\ mol}\right)$$

$$\times\left(\frac{4}{3}\pi\left(\frac{119nm}{2} - 3.7nm\right)^3\right) = 1.31\ proteins$$

Therefore, the 3 μM solution is expected to produce liposomes that contain an average of 1.3 caseins. Following a similar procedure, liposomes prepared using a solution of 200 nM ClpB are expected to have a λ of only 0.09, that is, less than 1 in 10 liposomes contain ClpB. While the above method is, of course, approximate, liposomes prepared from a solution containing both ClpB and casein are expected, on average, to be sparsely populated with ClpB and to contain only a single casein. We note that our smFRET experiments focus only on liposomes containing ClpB, and therefore, the excess of liposomes that do not contain ClpB are not monitored in our experiments.

Fluorescence anisotropy decay experiments. Solutions of the fluorescently labeled proteins (ca. 200 nM) in HEPES buffer were excited (510 nm pulsed laser diode) using vertically polarized light and monitored (570 nm, 16 nm slit width) along the vertical (I_{VV}) and horizontal (I_{VH}) axes on a FluoroHub time-correlated single-photon counting instrument (Horiba). Photons were collected in 4096 channels with a time-per-channel of 13.65 ps. Following a previously reported procedure (7), the fluorescence anisotropy was quantified by globally analyzing I_{VV} and I_{VH} according to Equations S1 – S2 using an in-house Matlab script.

$$I_{vv}(t) = IRF * \left(\frac{I(t)}{3} \left(1 + 2r(t)\right) \right) \tag{1}$$

$$I_{vh}(t) = IRF * \left(\frac{I(t)}{3G} \left(1 - r(t)\right) \right) \tag{2}$$

In Equations S1 and S2, IRF is the instrument response function, which was determined using a Ludox solution, * denotes the discrete convolution of the *IRF* with the model, the G-factor (G) is the deviation of the detectors from uniform detection of the two polarization, which was determined using Horiba software through integration of the I_{HV} and I_{HH} channels, $I(t)$ is the fluorescence decay of the dye in the absence of anisotropy, and r(t) is the time-dependent anisotropy given in Supplementary Equation 3. Supplementary Equation 3 assumes the fluorophore bound to the protein experiences segmental motion with a fast-tumbling time ϕ_F, while the protein experiences a slower tumbling time ϕ_P (8). The preexponential factor α is related to the extent of the hindrance of the fluorophore motion, and r_0 (= 0.40) is the limiting anisotropy at time zero.

$$r(t) = r_0 \left(\alpha \exp\left(-\frac{t}{\phi_F} \right) + (1 - \alpha) \right) \exp\left(-\frac{t}{\phi_P} \right) \tag{3}$$

The dye Cy3B required a single fluorescence lifetime (τ_D), as shown in Supplementary Equation 4.

$$I(t) = I_0 \exp\left(-\frac{t}{\tau_D} \right) \tag{4}$$

The parameters were optimized with the trust-region-reflective algorithm (9), and the uncertainties were estimated using nonlinear regression estimators. The goodness-of-fit was quantified using the normalized χ^2 term, where unity reflects a perfect fit with Poisson noise. A list of all retrieved parameters is given in Supplementary Tables 10 and 11. An example fit of a fluorescence decay is given in Supplementary Figure 30. The steady-state fluorescence anisotropy ($r_{SS,calc}$) was estimated from the retrieved parameters according to Supplementary Equation 5.

$$r_{SS} = \frac{\int_0^\infty r(t) I(t)\, dt}{\int_0^\infty I(t)\, dt} \tag{5}$$

Distribution of labeled casein inside liposomes. Proper interpretation of the smFRET results requires knowledge of the number of dyes covalently bound to casein. In the three-color experiments, casein was labeled with the donor, allowing us to directly observe the number of dyes inside the liposome by counting the number of donor photobleaching steps and selecting only liposomes that contained a single step. On the other hand, casein was labeled with the acceptor CF660R in the two-color experiments, making the number of photobleaching steps difficult to determine using continuous wave donor excitation. To minimize the probability of double-labeling casein, labeling was carried out with a sub-stoichiometric amount of dye of ca. 0.6 eq. Furthermore, to demonstrate that the majority of casein molecules were single labeled, the labeled casein was loaded into liposomes at the relatively low concentration of 1 μM (with respect to the dye), corresponding to an average of 0.44 (= [dye]×liposome volume) dyes per liposome. As the vesicles were formed, the casein molecules distributed themselves according to a Poisson distribution, with an expectation value equal to the average number of molecules per liposome. If casein contained a significant fraction of double-labeled molecules, the dye would not obey the same distribution, as there would be a significant increase in the occurrence of two or more acceptors per liposome. Supplementary Figure 6 demonstrates that the distribution of CF660R dyes obeys the Poisson distribution, as expected for single-labeled casein.

Derivation of leak and background corrections. While the correction of channel crosstalk is relatively straightforward for two-color FRET experiments, we derived general matrix-based equations for the crosstalk and background correction for an arbitrary number of fluorescent species. For the sake of simplicity, derivation is shown for the case of three dyes, as is the case for our three-color experiments. The final equation used is Supplementary Equation 11, which corrects all channels in a single operation.

Let O_i be the total observed fluorescence intensity in channel *i*, given by the fluorescence of each species in the channel, plus a background term.

$$O_i = \sum_j O_{i,j} + bg_i = \sum_j l_{i,j} \cdot F_{j,j} + bg_i \tag{6}$$

Where $O_{i,j}$ is the observed fluorescence of species *j* in channel *i*, bg_i is the background in channel *i*, $l_{i,j}$ is the leak factor of species *j* in channel *i* ($i_{j,j} = F_{i,j}/F_{j,j}$, and noting that $l_{j,j} = 1$), and $F_{i,j}$ is the fluorescence intensity of species *j* in channel *i*. In matrix notation, Supplementary Equation 6 becomes,

$$O = \begin{bmatrix} O_1 \\ O_2 \\ O_3 \end{bmatrix} = \begin{bmatrix} 1 & l_{1,2} & l_{1,3} \\ l_{2,1} & 1 & l_{2,3} \\ l_{3,1} & l_{3,2} & 1 \end{bmatrix} \begin{bmatrix} F_{1,1} \\ F_{2,2} \\ F_{3,3} \end{bmatrix} + \begin{bmatrix} bg_1 \\ bg_2 \\ bg_3 \end{bmatrix} = l \times f + BG \tag{7}$$

Where O is the matrix of observed fluorescence in each channel, l is the matrix of leak values, f is the vector of $F_{j,j}$ values, and BG is the vector of background values in each channel. The total fluorescence of species j is given by the sum of its contributions to each channel.

$$F_j = \sum_i F_{i,j} = \sum_i l_{i,j} \cdot F_{j,j} = F_{j,j} \cdot \sum_i l_{i,j} \tag{8}$$

In matrix notation,

$$F = \begin{bmatrix} F_1 \\ F_2 \\ F_3 \end{bmatrix} = \left(\begin{bmatrix} 1 & 1 & 1 \end{bmatrix} \begin{bmatrix} 1 & l_{1,2} & l_{1,3} \\ l_{2,1} & 1 & l_{2,3} \\ l_{3,1} & l_{3,2} & 1 \end{bmatrix} \right)^T \circ \begin{bmatrix} F_{1,1} \\ F_{2,2} \\ F_{3,3} \end{bmatrix} = L \circ f \tag{9}$$

where ○ denotes the element-wise product and L is given by,

$$L = \left(\begin{bmatrix} 1 & 1 & 1 \end{bmatrix} \begin{bmatrix} 1 & l_{1,2} & l_{1,3} \\ l_{2,1} & 1 & l_{2,3} \\ l_{3,1} & l_{3,2} & 1 \end{bmatrix} \right)^T = \begin{bmatrix} 1 & l_{1,2} & l_{1,3} \\ l_{2,1} & 1 & l_{2,3} \\ l_{3,1} & l_{3,2} & 1 \end{bmatrix}^T \begin{bmatrix} 1 \\ 1 \\ 1 \end{bmatrix} \tag{10}$$

Substituting f in (S7) into (S9) yields the corrected fluorescence as S11.

$$F = L \circ l^{-1} \left(O - BG \right) \tag{11}$$

Leak factor determination. The leak factors l_j for each species j are determined by acquiring fluorescence from single-labeled molecules. The matrix l is decomposed into separate elements for each species j.

$$l = \begin{bmatrix} 1 & l_{1,2} & l_{1,3} \\ l_{2,1} & 1 & l_{2,3} \\ l_{3,1} & l_{3,2} & 1 \end{bmatrix} = \begin{bmatrix} l_1 & l_2 & l_3 \end{bmatrix} \tag{12}$$

Rearranging Equation (S7), we know,

$$\begin{bmatrix} O_1 \\ O_2 \\ O_3 \end{bmatrix} - \begin{bmatrix} bg_1 \\ bg_2 \\ bg_3 \end{bmatrix} = \begin{bmatrix} 1 & l_{1,2} & l_{1,3} \\ l_{2,1} & 1 & l_{2,3} \\ l_{3,1} & l_{3,2} & 1 \end{bmatrix} \begin{bmatrix} F_{1,1} \\ F_{2,2} \\ F_{3,3} \end{bmatrix} \tag{13}$$

Given that for a single-labeled molecule, the only contribution to each channel arises from the fluorescence of species j, the corresponding l_j element of the leak matrix can be found using Supplementary Equation 14.

$$l_j = \frac{\begin{bmatrix} O_1 \\ O_2 \\ O_3 \end{bmatrix} - \begin{bmatrix} bg_1 \\ bg_2 \\ bg_3 \end{bmatrix}}{F_{j,j}} = \frac{O - BG}{F_{j,j}} = \frac{O - BG}{O_j - bg_j} \tag{14}$$

A plot of the molecule-averaged leak factors for Cy3B in the two-color experiments is given in Supplementary Figure 31. The emission of CF660R was sufficiently separated that its emission in the Cy3B channel was negligible (i.e., leak factor = 0). The corresponding leak factor matrix is given by $\begin{bmatrix} 1 & 0 \\ 0.148 & 1 \end{bmatrix}$. Supplementary Figure 32 plots the leak factors for Cy3B, AF647, and CF680R in the 3-color experiments. The corresponding leak factor matrix is given by $l = \begin{bmatrix} 1 & 0.01 & 0.00 \\ 0.07 & 1 & 0.11 \\ 0.04 & 0.61 & 1 \end{bmatrix}$.

Determining inter-acceptor energy transfer: The presence of FRET between the two acceptors (with a separation of 6.8 nm) in the three-color experiments can potentially affect the interpretation of the results. The fluorescence of double-labeled ClpB molecules was collected after acceptor excitation to assess the extent of inter-acceptor FRET. After leak correction, the presence of inter-acceptor energy transfer was assessed by comparing the photon flux of acceptor 2 (CF680R, the more red-shifted dye) before and after photobleaching of acceptor 1 (AF647). When energy transfer occurs between the dyes (from A1 to A2), the fluorescence intensity of A2 would be larger before the photobleaching of A1. However, as shown in Supplementary Figure 18, the absence of such an effect indicates the lack of energy transfer between acceptors.

Fluorescence correlation spectroscopy. Data was collected using continuous-wave excitation (560 nm, 30 μW). The fluorescence emission was split with a 50/50 beam-splitting cube and recorded on two detectors to avoid after-pulsing effects. FCS curves were generated using the PicoQuant SymPhoTime 64 software (Supplementary Figure 27). The curves were fitted to Supplementary Equation 15 below using a weighted least-squares function, where the weight of each point was determined by its standard deviation. In this equation, ω is the aspect ratio of the confocal volume, N is the average number of molecules in the confocal volume, τ_D is the diffusion time of a molecule through the confocal volume, τ_T is the triplet lifetime, f_T is the fraction of triplet state, and G_∞ is the autocorrelation at a very long time. The retrieved parameters are provided in Supplementary Figure 12.

$$G(t) = \frac{1}{N}\left(1 + \frac{t}{\tau_D}\right)^{-1}\left(1 + \frac{t}{\omega^2 \tau_D}\right)^{-\frac{1}{2}}\left(1 + \frac{f_T}{1 - f_T} e^{\frac{-t}{\tau_T}}\right) + G_\infty \tag{15}$$

Using the known diffusion coefficient of Cy3B (D_0 = 334 μm²/s at T_0 = 24 °C) (10, 11) and a confocal volume (V_{eff}) of 0.286 fL (calculated using the known D_0), the temperature T in the

confocal volume was calculated using Supplementary Equation 16, where the viscosity (η) was corrected using the Vogel Relation given in Supplementary Equation 17. In Supplementary Equation 17, temperature (T) is in K, and the empirical constants a, b, and c are equal to –4.5318, –220.57, and 149.39, respectively (12).

$$\frac{T}{\eta} = \frac{V_{eff}^{\frac{2}{3}}}{4\pi\omega^{\frac{2}{3}}\tau_D} \frac{T_0}{D_0\eta_0} \tag{16}$$

$$\log(\eta) = a - \frac{b}{T - c} \tag{17}$$

Supplementary Equation 16 is derived starting from the relationship between effective confocal volume (V_{eff}) and D given in Supplementary Equation 18 (13). The change in diffusion coefficient with temperature (Supplementary Equation 19) is determined using the Stokes-Einstein equation for diffusion. D in Supplementary Equation 18 was substituted into S19, which, after rearrangement, yielded S16.

$$V_{eff} = \pi^{3/2}\omega\left(4D\tau_D\right)^{3/2} \tag{18}$$

$$\frac{D}{D_0} = \frac{T}{\eta}\frac{\eta_0}{T_0} \tag{19}$$

Supplementary Figures

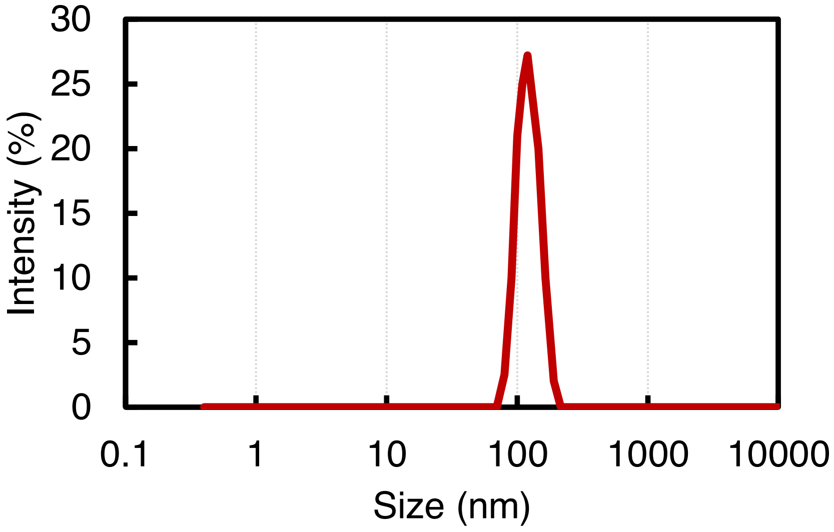

Supplementary Figure 1. Extrusion produces monodisperse large unilamellar vesicles. Sample distribution of the sizes of 99:1 DMPC: Biotinyl PE liposomes prepared by extrusion through a 0.1 µm membrane, as measured by dynamic light scattering (DLS, Malvern Zetasizer). Extrusion produced vesicles of a unimodal distribution with a *z*-average size of 119 ± 1 nm and a dispersity of 0.05 ± 0.01. Measurements were repeated in triplicate, see Supplementary Figure 9.

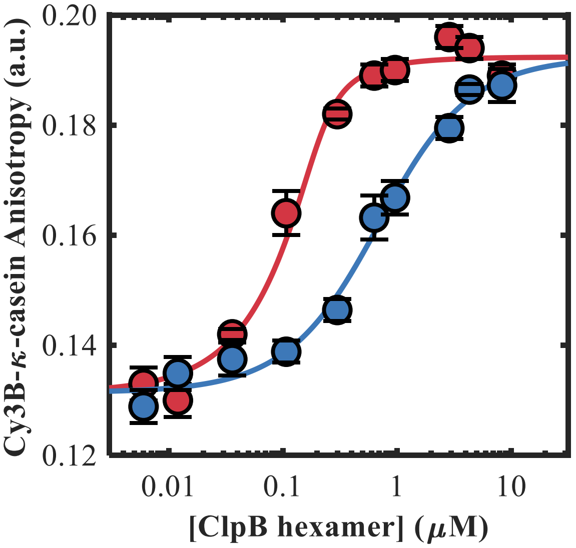

Supplementary Figure 2. ATPγS increases the apparent affinity of casein to ClpB. Steady-state anisotropy of Cy3B labeled κ-casein (200 nM) in the presence of increasing concentrations of WT ClpB, as well as (blue) 2 mM ATP or (red) 2 mM ATPγS. All measurements were repeated in triplicate. The anisotropy values were fitted (lines) with a bimolecular binding equilibrium curve, yielding apparent dissociation constants of 600 ± 100 nM and 20 ± 10 nM in the presence of ATP and ATPγS, respectively. The apparent constants are affected by the different times of interaction between casein and ClpB in the presence of ATP or ATPγS; smFRET experiments revealed a similar 34-fold increase in dwell time in the presence of ATPγS. Data is presented as mean +/- SD.

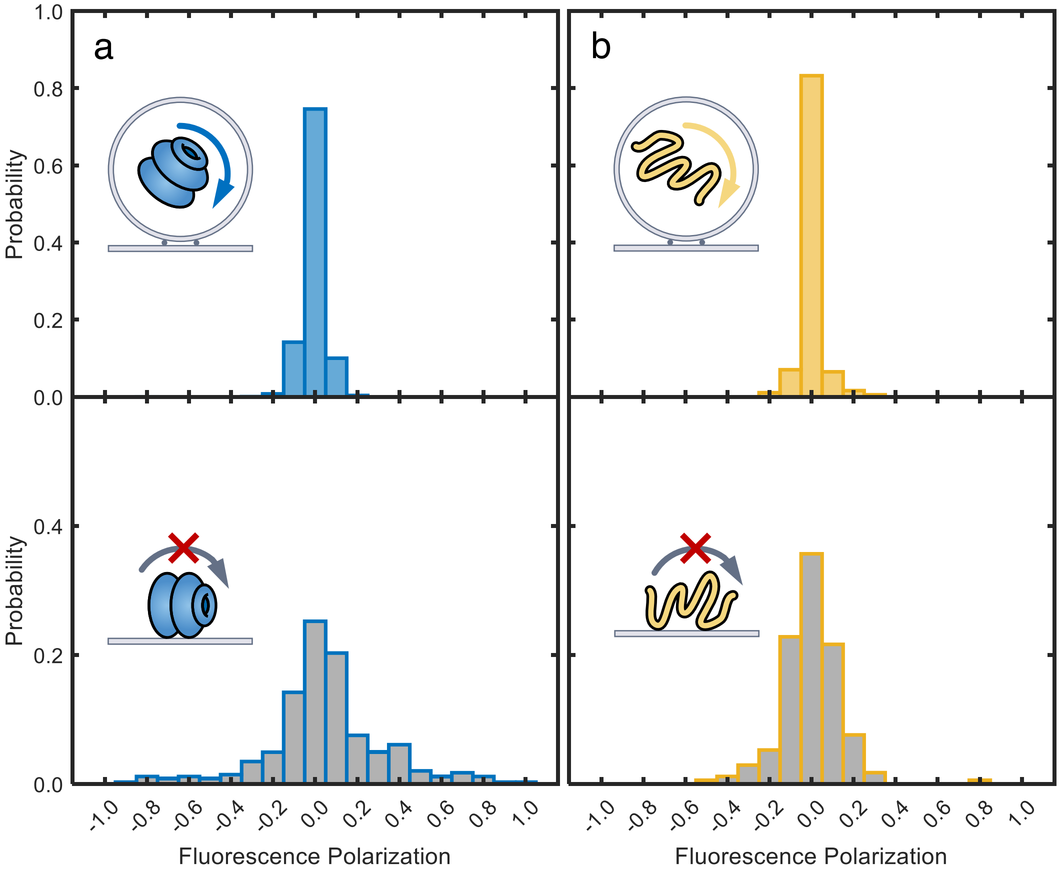

Supplementary Figure 3. ClpB and κ-casein freely diffuse inside immobilized liposomes. Distributions of the fluorescence polarization (P = $(I_V – I_H)/(I_V + I_H)$) of fluorescently labeled (**a**) ClpB (359C, Cy3B) and (**b**) κ-casein (sigma, CF660R) trapped inside surface-immobilized DMPC liposomes (blue) and directly immobilized on a glass surface (grey). The polarization distribution for ClpB ($\sigma = 0.05$, $N = 185$ molecules) and casein ($\sigma = 0.07$, $N = 153$) inside the vesicles is much narrower than for protein molecules non-specifically immobilized on a glass surface (ClpB: $\sigma = 0.14$, $N = 463$, casein: $\sigma = 0.35$, $N = 342$). The narrow distribution demonstrates that ClpB and casein have rotational freedom and can freely diffuse within the vesicles.

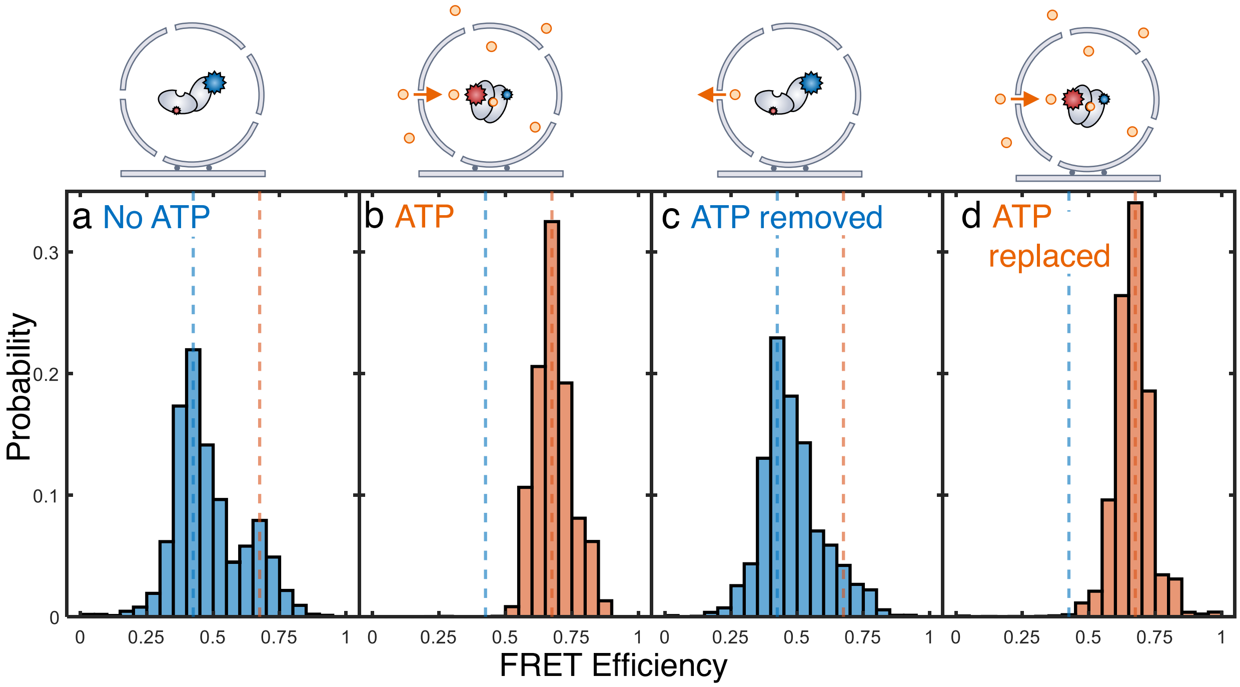

Supplementary Figure 4. ATP readily diffuses in and out of DMPC liposomes. Histograms of the FRET efficiency values of fluorescently-labeled adenylate kinase variant AK82V (3) encapsulated in surface-immobilized DMPC (14:0 PC) liposomes at 22.5 °C without (**a**,**c**) and with (**b**,**d**) 2 mM ATP. (**a**) The protein was encapsulated without ATP (50 mM TRIS, 100 mM KCl, 15 mM $MgCl_2$, pH = 8) and the vesicles were immobilized onto a glass surface inside a flow cell. The histogram (N = 116 molecules) displays a major population at low FRET efficiency (ca. 0.4), as expected for apo AK (3). (**b**) Upon the addition of 2 mM ATP, the entire population (N = 97) shifted to a high FRET efficiency (ca. 0.68), demonstrating that ATP could permeate through the DMPC membranes and reach the encapsulated protein. (**c**) The reversibility of the process was demonstrated by the recovery of the low FRET population (N = 55) when the cell was flushed with buffer without ATP. (**d**) The high FRET population (N = 65) reappeared upon the repeated addition of ATP.

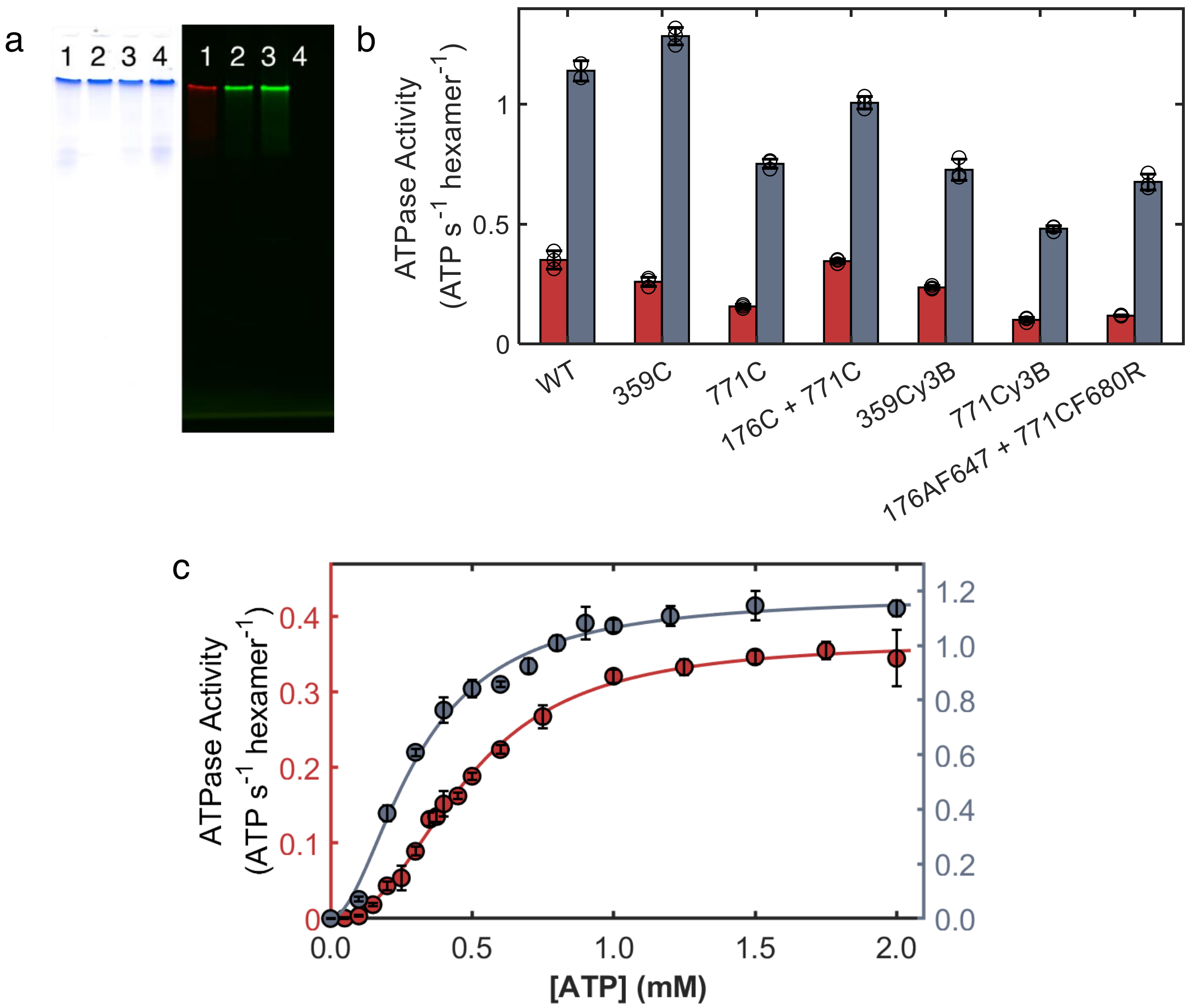

Supplementary Figure 5. ClpB remains active after point mutations and fluorescence labeling. (**a**) Native gel electrophoresis (4 % acrylamide, with 10 mM $MgCl_2$, 2 mM ATP at 70 V for 12 hours (4)) of reassembled fluorescently labeled double cysteine mutant ClpB (176 – AF647 + 771 – CF680R, lane 1), single cysteine mutants labeled with Cy3B (359, lane 2 and 771, lane 3), and wild-type (WT) ClpB (lane 4). Shown are the Coomassie Blue stain (left) and fluorescence (Typhoon scanner, right) following excitation at 635 nm (red) and 532 nm (green). As previously reported (4, 14), the presence of a high molecular weight single band demonstrates the successful hexameric assembly. The red dyes on the double-labeled ClpB exhibited fluorescence under red light, both ClpB variants labeled with Cy3B exhibited fluorescence under green light, while the WT ClpB experienced no fluorescence. (**b**) ATPase activity of ClpB at 22.5 °C in the presence of 2 mM ATP (red) and upon addition of 3 μM κ-casein (grey). The activity is shown for the WT ClpB, single cysteine mutants (359C, 771C), double mutant (176C + 771), and the fully-fluorescently labeled counterparts. (**c**) ATPase activity of WT ClpB as a function of ATP concentration without (red, left axis) and with (grey, right axis) the addition of 3 μM κ-casein. Data is presented as mean +/- SD of triplicates. The solid lines represent the fits according to the Hill equation with Hill coefficients of 2.7 ± 0.1 and 1.9 ± 0.2 and K_m values of 0.42 ± 0.01 mM and 0.31 ± 0.02 mM, respectively. Activity values are similar to literature-reported values (4, 15).

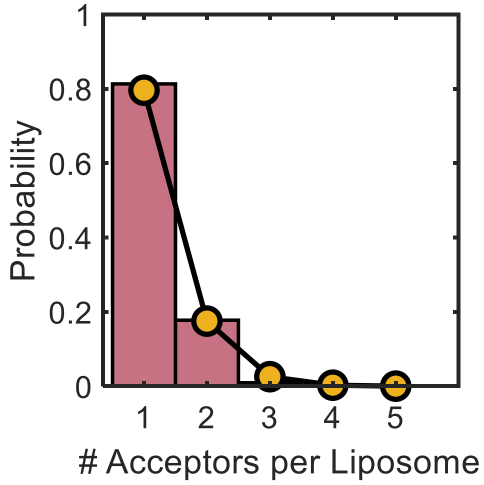

Supplementary Figure 6. Photobleaching experiments on CF660R labeled casein demonstrate casein overwhelmingly contains at most a single acceptor. Histogram (red) of the number of photobleaching steps for 107 liposomes loaded with 1 μM CF660R-labeled casein, corresponding to an average number of dyes per liposome of 0.44. The orange points represent the expected probability based on the Poisson distribution for single-labeled casein, Pr(# acceptors) = $\Pr(i \mid i > 0) = \text{Pois}(i, \lambda)/(1 - \text{Pois}(0, \lambda)$, where Pois is the Poisson distribution, $\lambda = 0.44$, and i (≥ 1) is the number of acceptors. The agreement between the Poisson distribution and the photobleaching experiments demonstrates that the bulk of casein cannot contain more than one acceptor. If casein contained a significant fraction of double-labeled molecules there would be a significant increase in the occurrence of two or more acceptors per liposome.

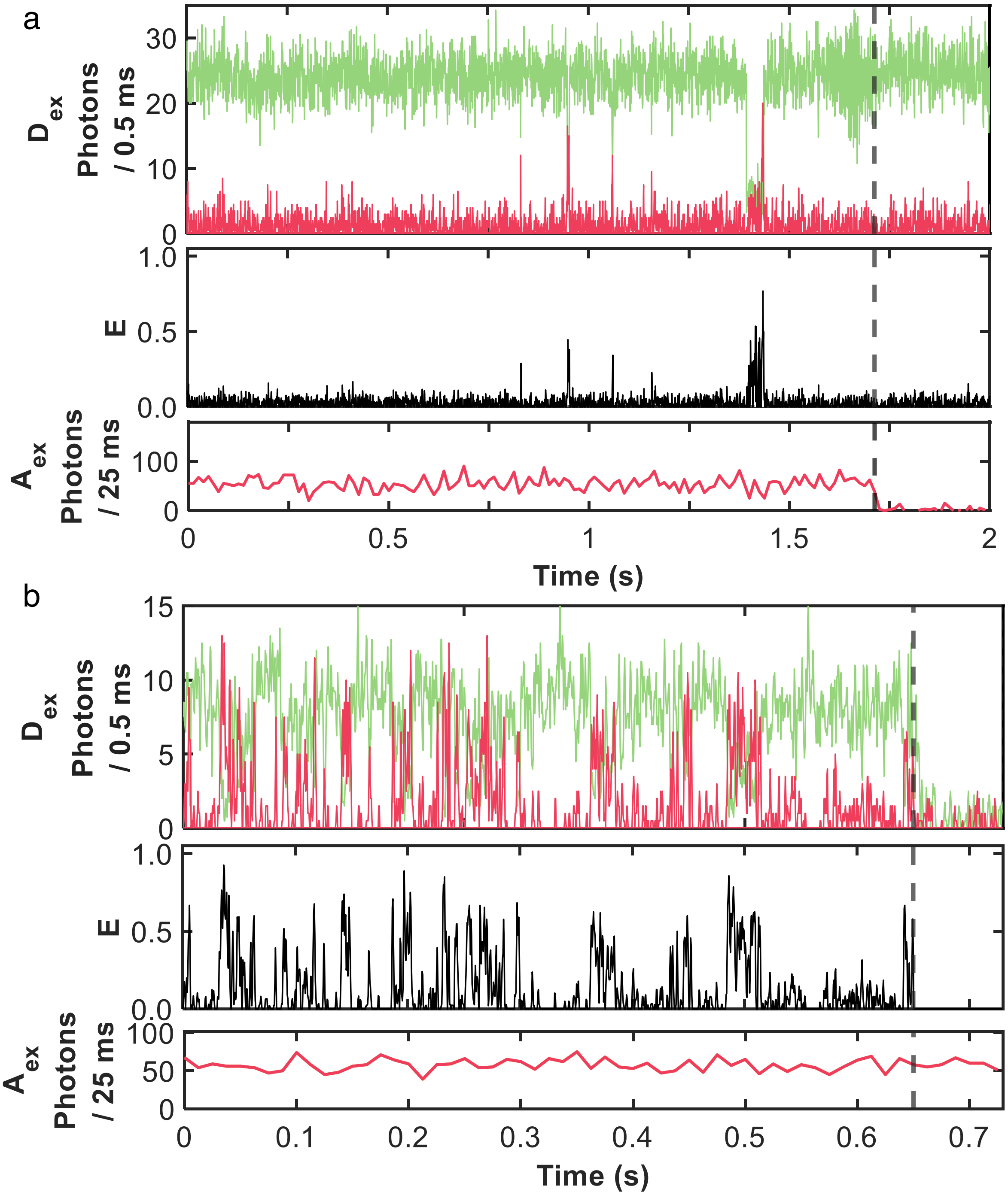

Supplementary Figure 7. The acceptor remains fluorescent throughout the trajectory. Sample trajectories of translocation of casein through (**a**) NBD1 (N = 27 molecules) and (**b**) NBD2 (N = 34 molecules) labeled ClpB using pulsed interleaved excitation (500 nW donor, 100 nW acceptor, 2 – 3 donor pulses followed by one acceptor pulse with intervals of 50 ns). Shown are the fluorescence after donor excitation (D_{ex}, top) of the donor (green) and acceptor (red), the apparent FRET efficiency (middle), and the acceptor fluorescence after direct acceptor excitation (A_{ex}, bottom). The dashed line marks the photobleaching of the (**a**) acceptor and (**b**) the donor. The constant acceptor signal (before photobleaching) demonstrates the acceptor remains fluorescent throughout the trajectory.

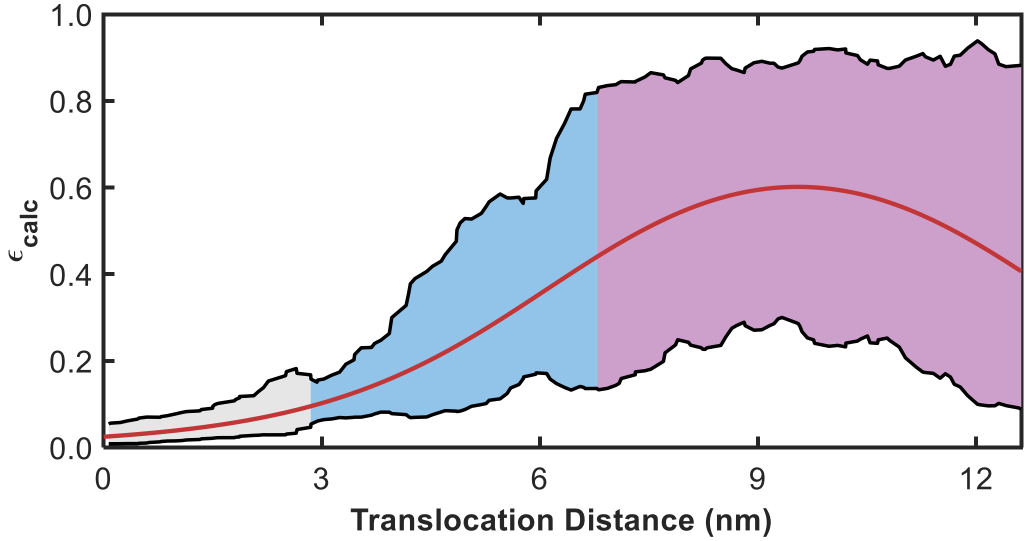

Supplementary Figure 8. Calculated FRET efficiencies for NBD2 labeled ClpB demonstrate that high FRET efficiency events can only occur if casein passes through the NBD2 ring. A map of the expected FRET efficiency (ε_{calc}) for an acceptor as it passes through ClpB with a donor located in the NBD2 (res. 771). The solid red line represents the centerline passing directly through the pore, while the black lines represent the boundaries of the pore. The shaded regions represent the *N*-terminal domain (NTD, grey) and the two NBDs (blue and purple).

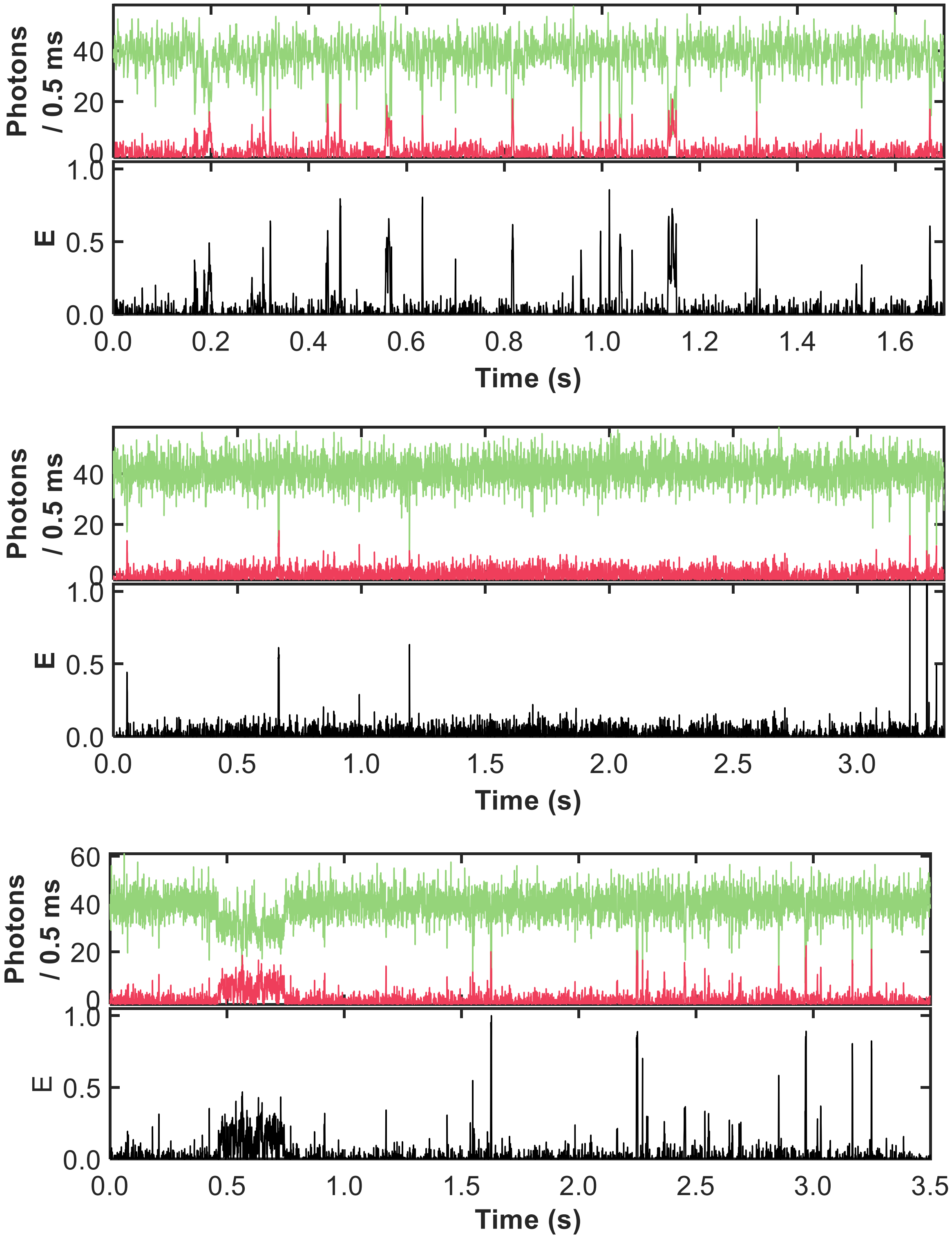

Supplementary Figure 9. Sample trajectories of translocation of casein through NBD1 labeled ClpB in the presence of 2 mM ATP. Shown are the fluorescence (top panels) of the donor (green) and acceptor (red) and (bottom panels, black) the FRET efficiency.

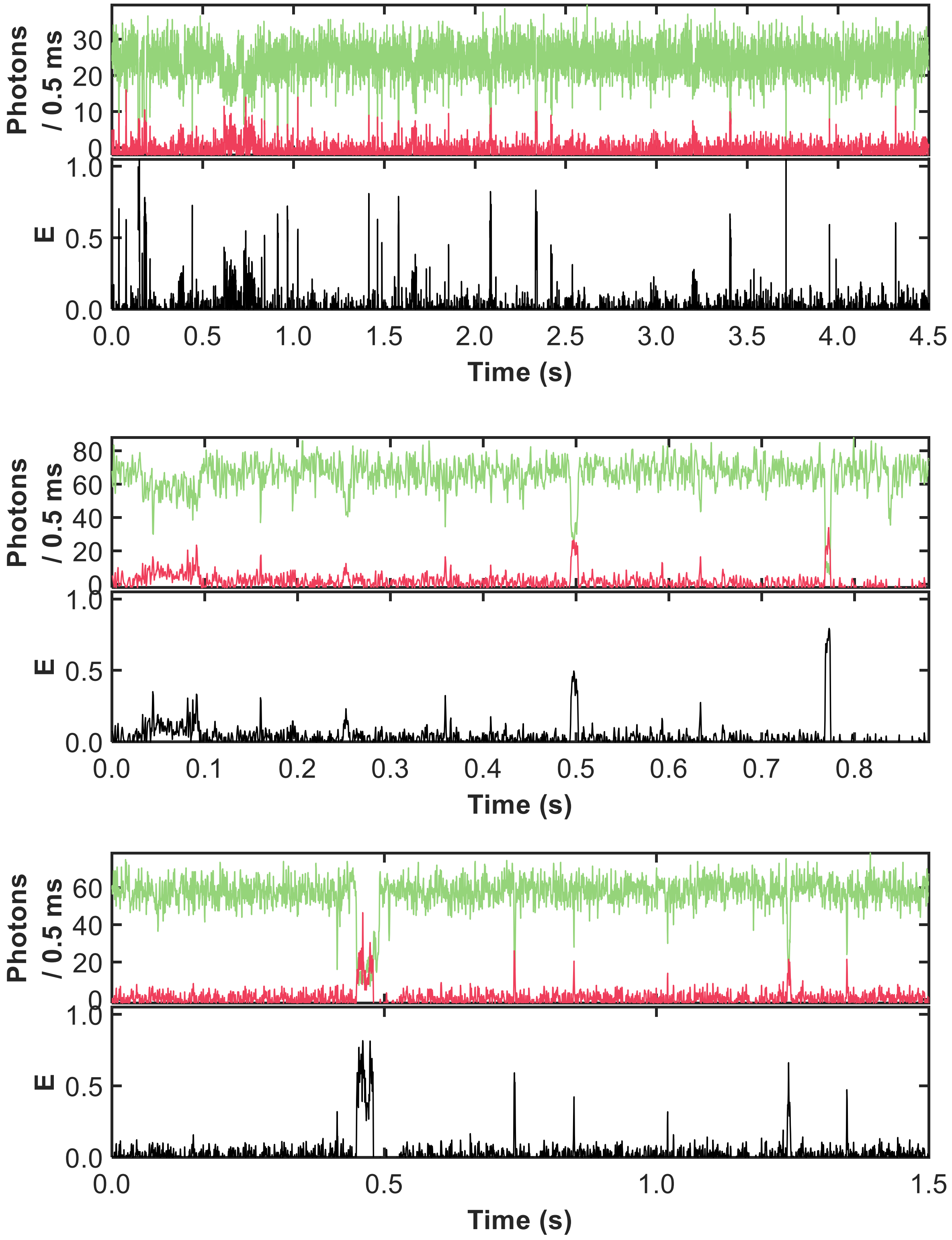

Supplementary Figure 10. Sample trajectories of translocation of casein through NBD2 labeled ClpB in the presence of 2 mM ATP. Shown are the fluorescence (top panels) of the donor (green) and acceptor (red) and (bottom panels, black) the FRET efficiency.

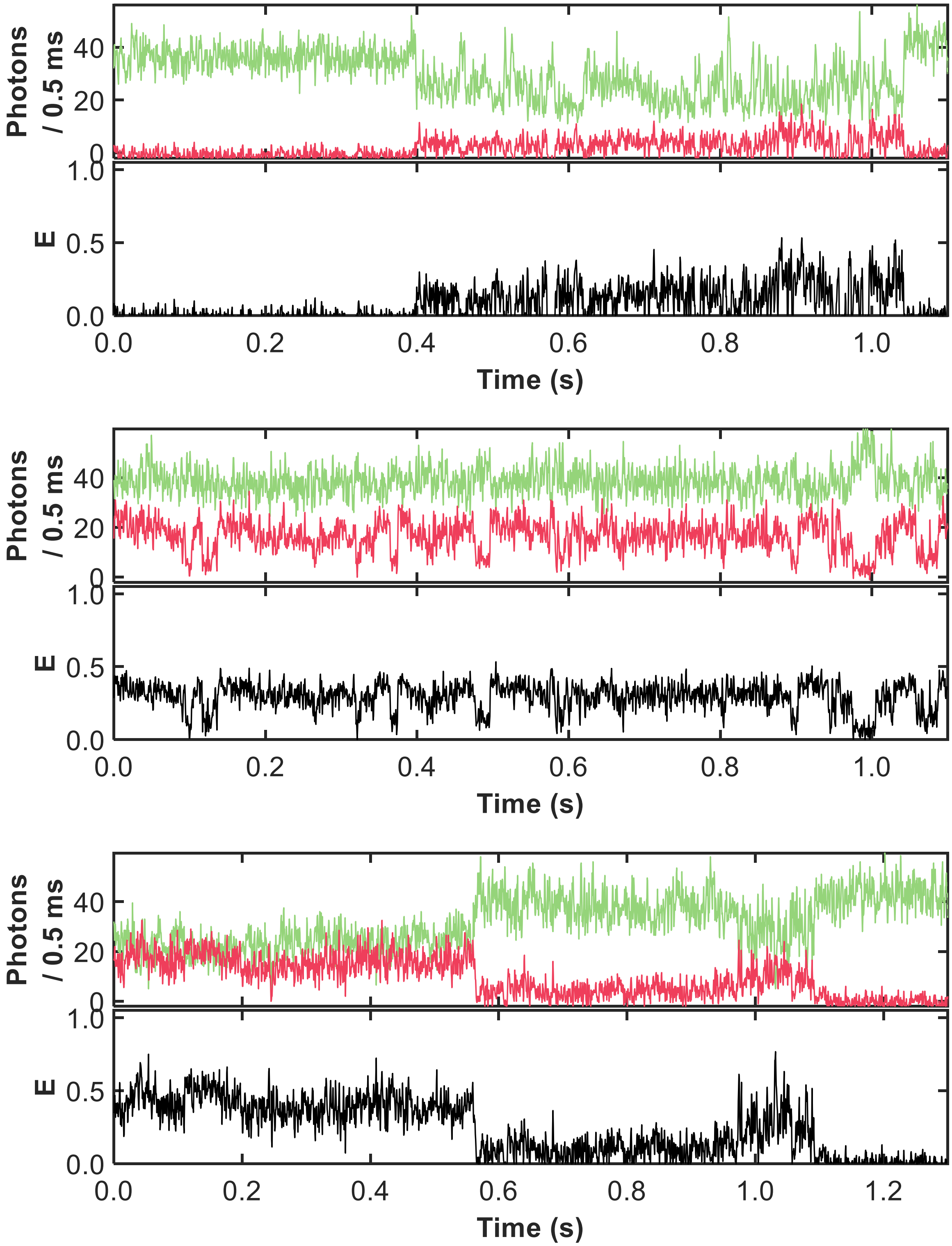

Supplementary Figure 11. Sample trajectories of translocation of casein through NBD1 labeled ClpB in the presence of 2 mM ATPγS. Shown are the fluorescence (top panels) of the donor (green) and acceptor (red) and (bottom panels, black) the FRET efficiency.

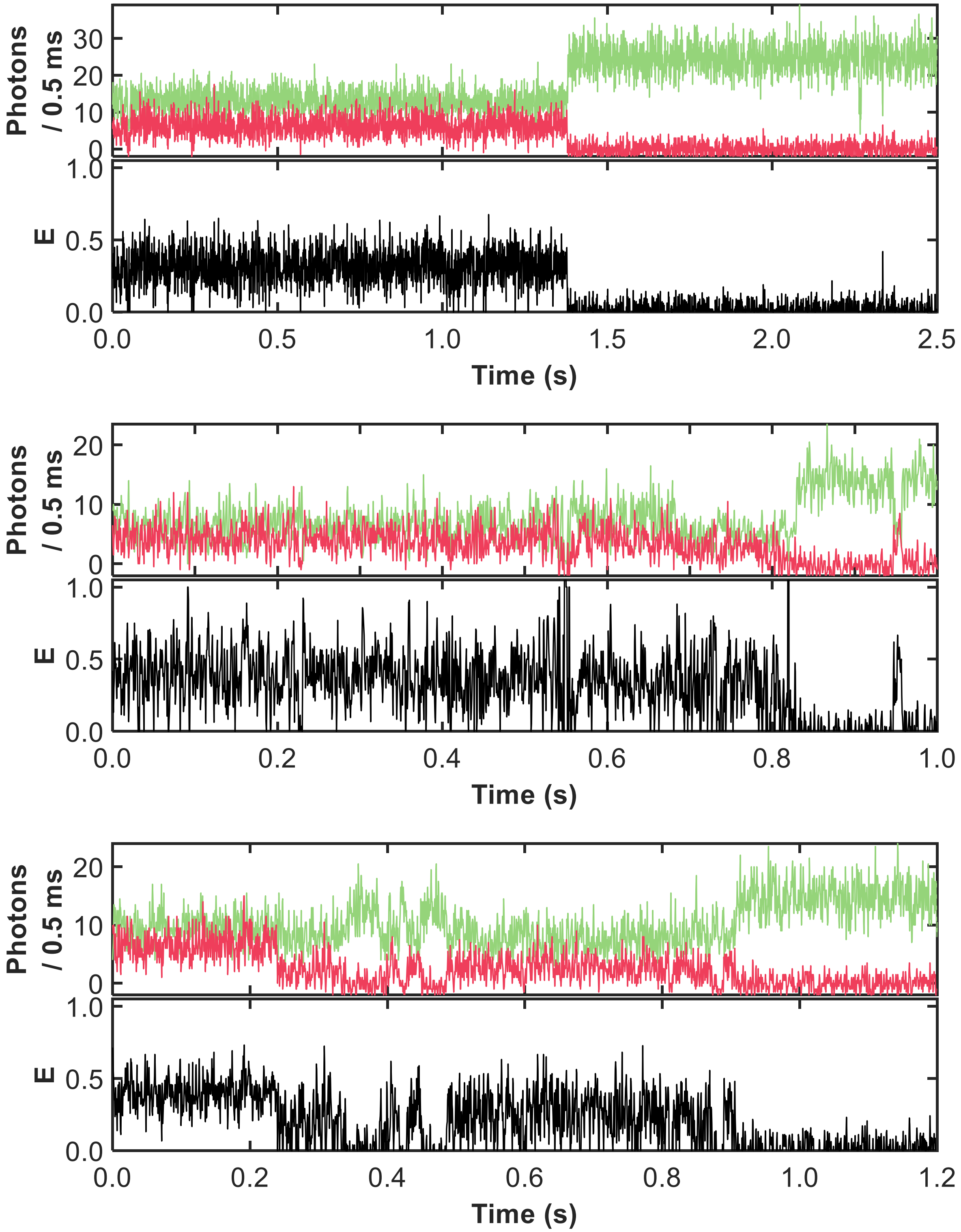

Supplementary Figure 12. Sample trajectories of translocation of casein through NBD2 labeled ClpB in the presence of 2 mM ATPγS. Shown are the fluorescence (top panels) of the donor (green) and acceptor (red) and (bottom panels, black) the FRET efficiency.

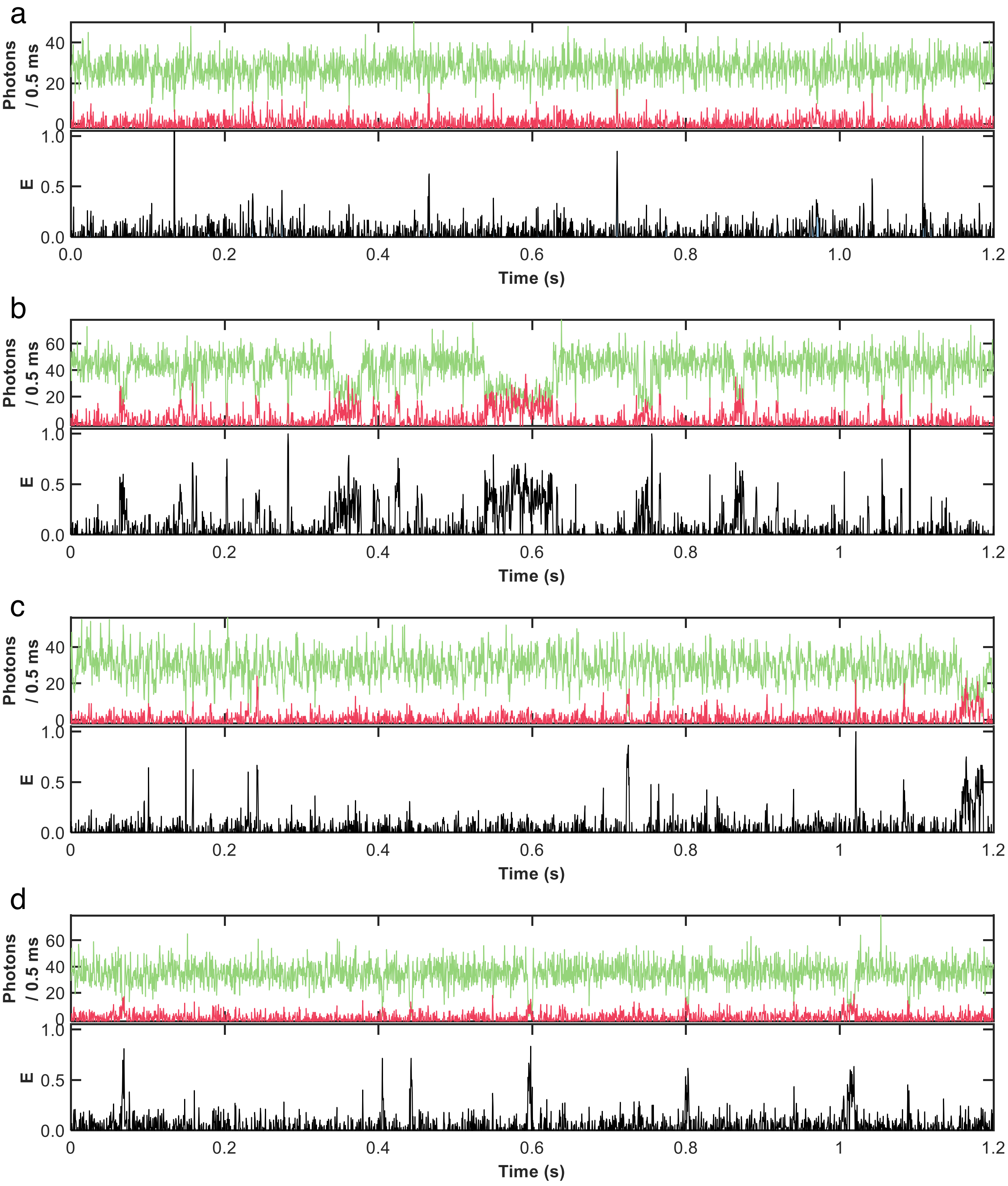

Supplementary Figure 13. Sample trajectories of translocation of casein as a function of temperature. Shown are the fluorescence (top panels) of the donor (green) and acceptor (red) and (bottom panels, black) the FRET efficiency of translocation events through (**a**,**b**) NBD1 and (**c**,**d**) NBD2 labeled ClpB at (**a**,**c**) 32 and (**b**,**d**) 10 °C.

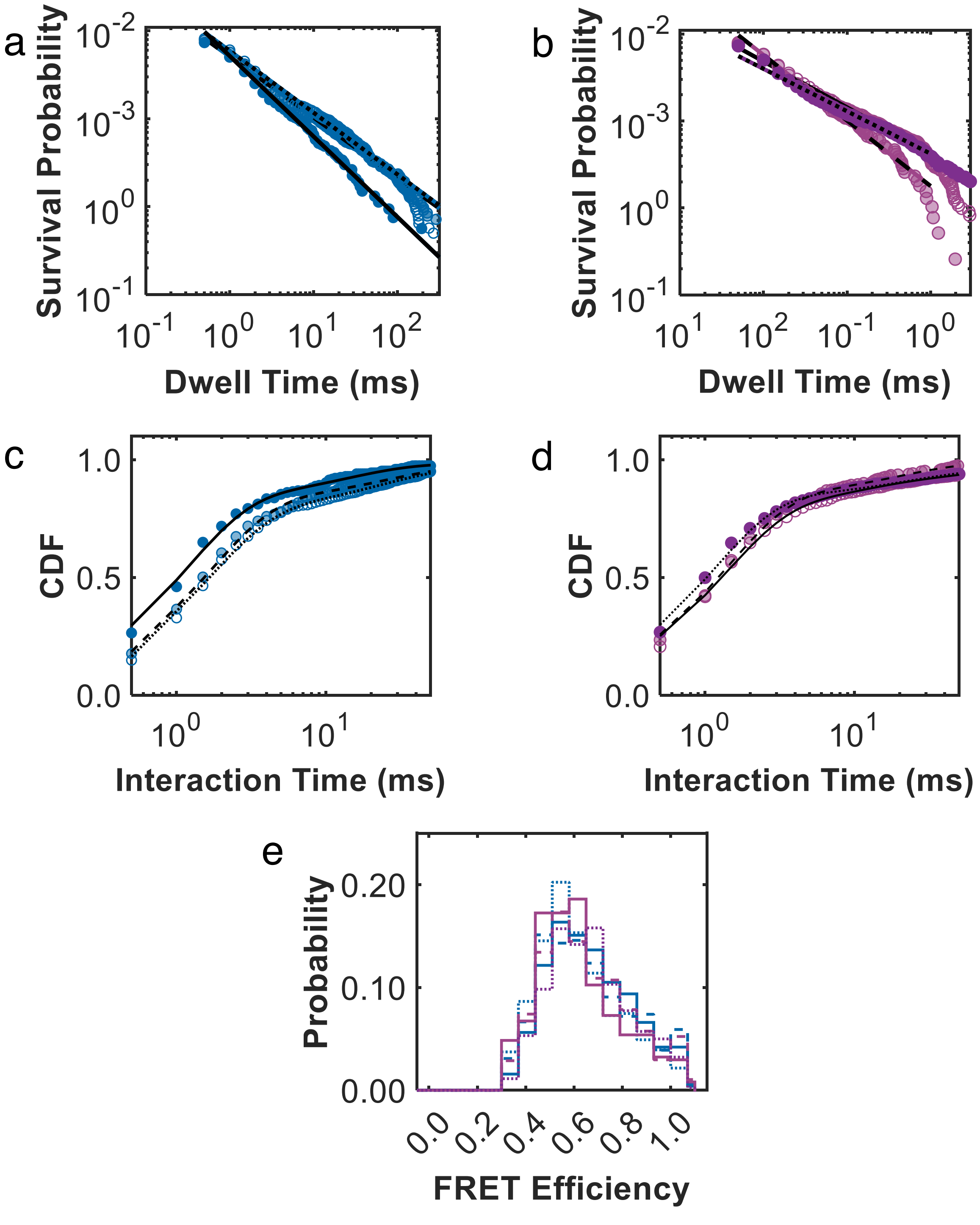

Supplementary Figure 14: Event dwell time is weakly affected by temperature. (**a**,**b**) Survival probability and (**c**,**d**) cumulative distribution function (CDF) of the dwell time and (**e**) peak FRET efficiency for NBD1 (blue) and NBD2 (purple) labeled ClpB at temperatures of 32 (empty symbols, dotted lines), 22.5 (shaded symbols, dashed lines), and 10 °C (filled symbols, solid lines). The parameters retrieved from the (**a**,**b**) power-law and (**c**,**d**) exponential fits are provided in Supplementary Tables 4 and 7. Statistics are provided in Supplementary Table 1.

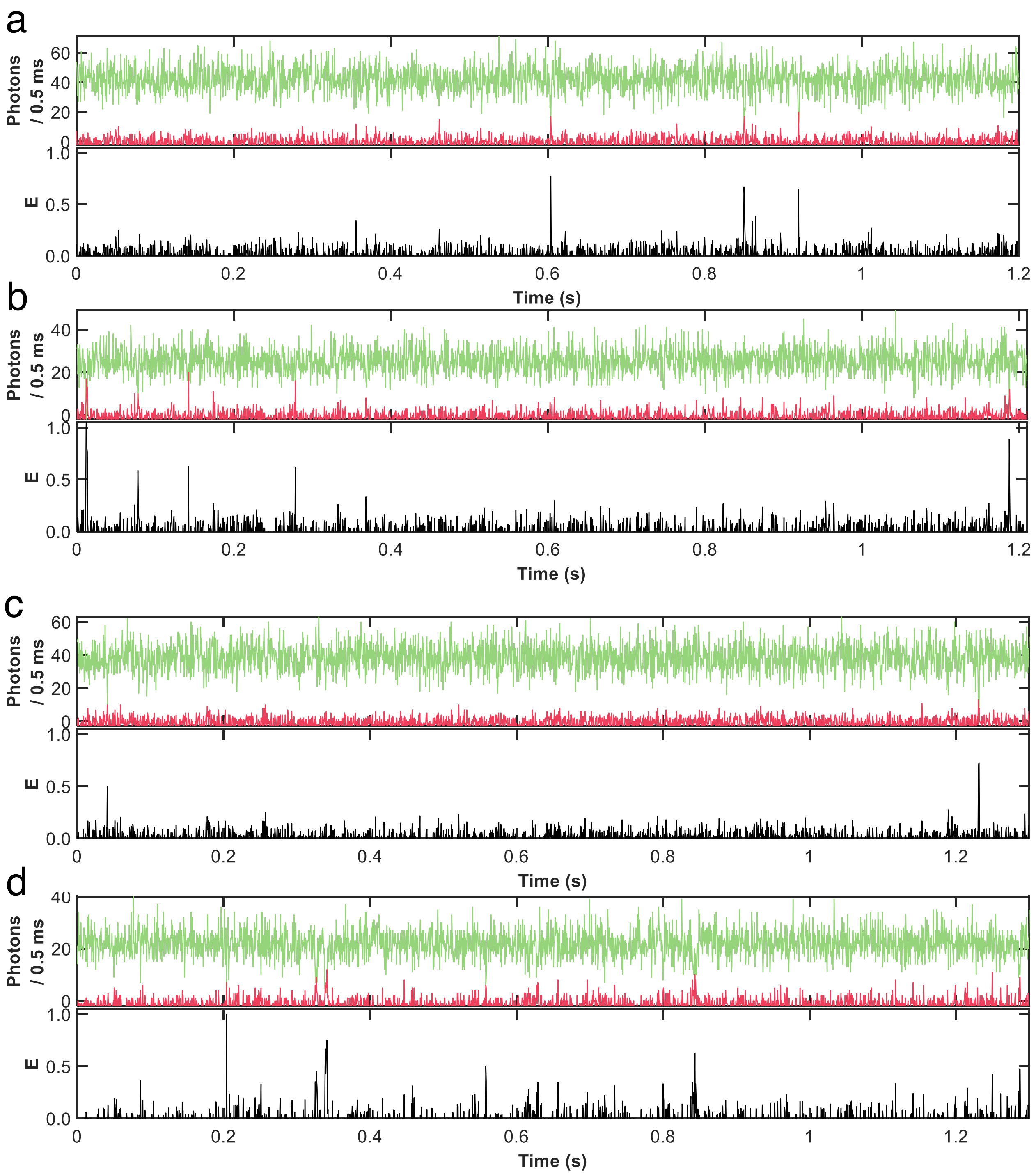

Supplementary Figure 15. Sample trajectories of translocation of casein as a function of ATP concentration. Shown are the fluorescence (top panels) of the donor (green) and acceptor (red) and (bottom panels, black) the FRET efficiency of translocation events through (**a**,**b**) NBD1 and (**c**,**d**) NBD2 labeled ClpB in the presence of (**a**,**c**) 0.2 and (**b**,**d**) 0.4 mM ATP.

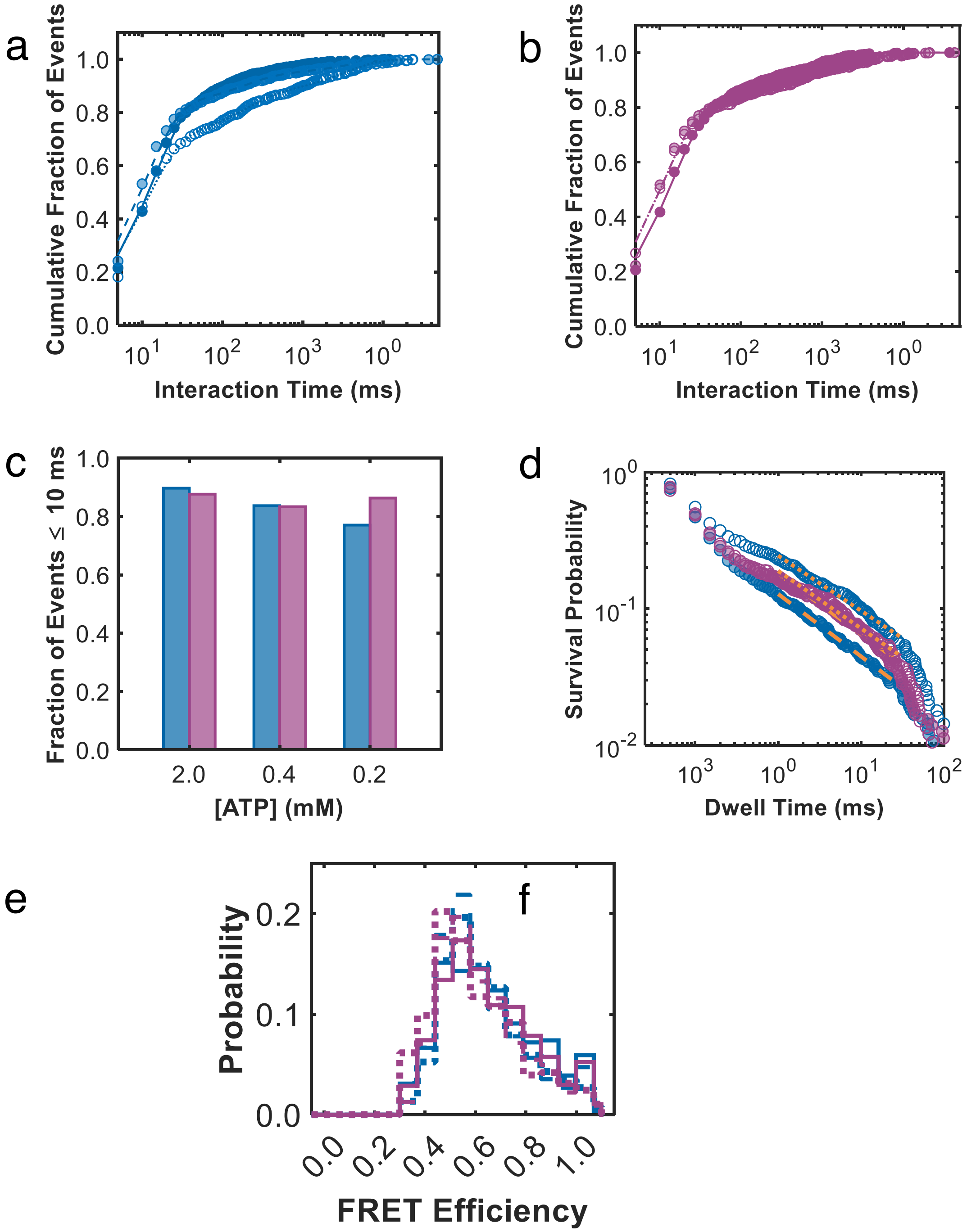

Supplementary Figure 16. Event dwell time is weakly affected by ATP concentration. (**a**,**b**) Cumulative distribution of event times, (**c**) fraction of short events, (**d**) power-law distribution, and (**e**) distribution of peak FRET efficiencies at ATP concentrations of 2.0 (solid lines, filled symbols), 0.4 (shaded symbols, dashed lines), and 0.2 mM (hollow symbols, dotted lines) for ClpB labeled on NBD1 (blue) and NBD2 (purple). Statistics are provided in Supplementary Table 1. The lines in (**a**,**b**,**d**) represent fits, whose parameters are reported in Supplementary Tables 4, 5, and 7.

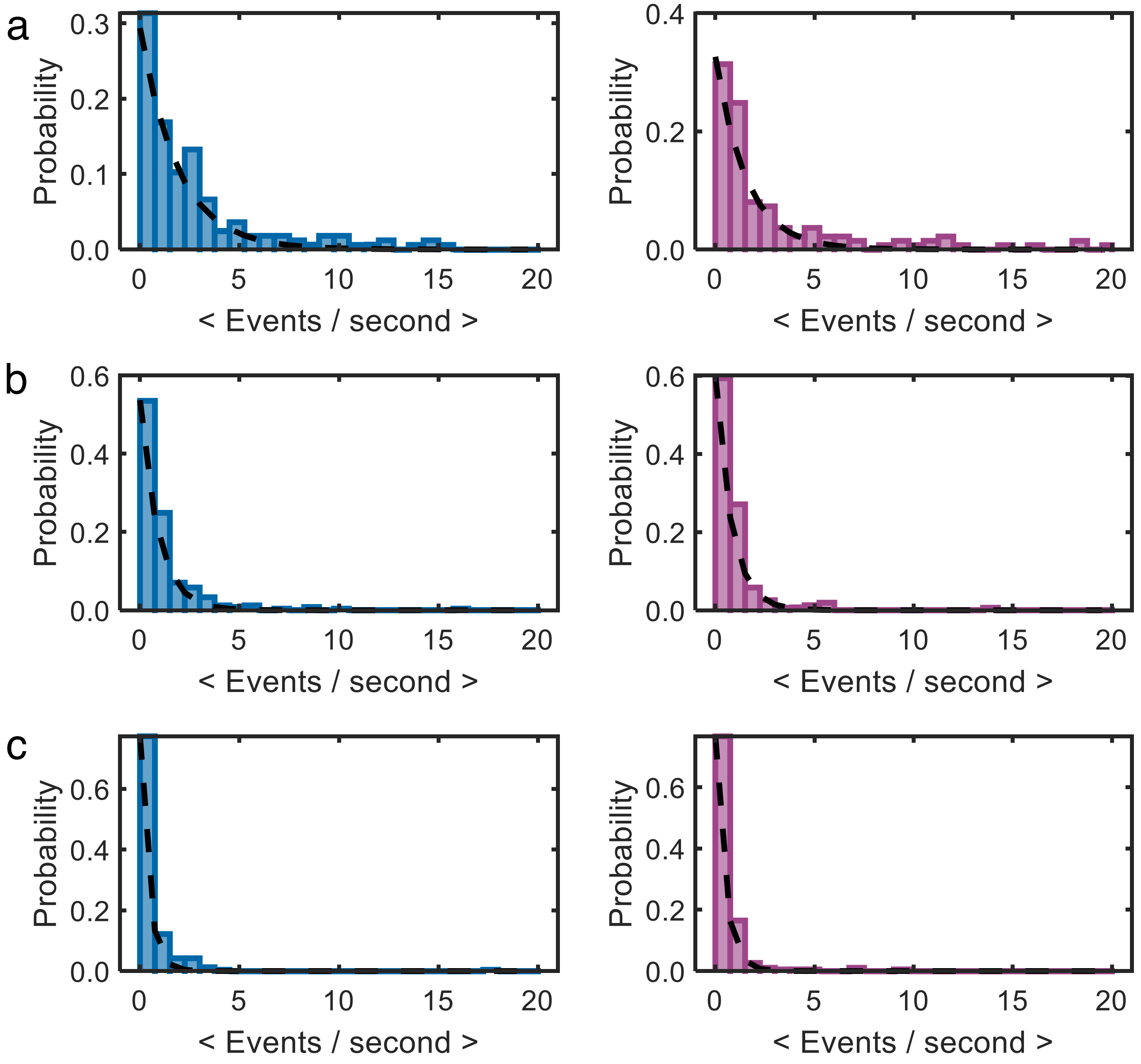

Supplementary Figure 17. Event frequency directly correlates with ATP concentration. Histograms of the average number of events per second for individual ClpB molecules at ATP concentrations of (**a**) 2.0, (**b**) 0.4, (**c**) 0.2 mM ATP for ClpB labeled on NBD1 (left, blue) and NBD2 (right, purple). The dashed lines represent the exponential fits of the distributions. Statistics are provided in Supplementary Table 1. The parameters retrieved from the fits are reported in Supplementary Figure 8 and in Figure 3b (in the main text).

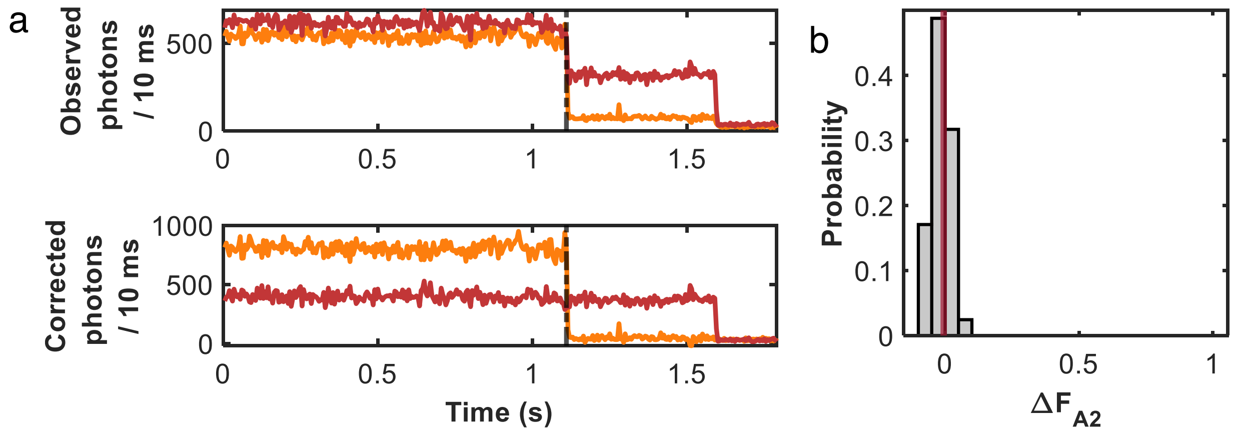

Supplementary Figure 18: Acceptor photobleaching study on double-acceptor labeled ClpB demonstrates negligible inter-acceptor energy transfer. (**a**) Sample trajectory of a double-labeled ClpB molecule after excitation at 640 nm. Both acceptor 1 (A1, orange) and acceptor 2 (A2, red) were excited by the 640 nm laser. The top panel displays the uncorrected trajectory and the bottom the leak-corrected trajectory (See *Leak Factor Determination*). The black dashed line indicates the photobleaching of A1. Note the unchanged intensity of A2 in the lower panel, indicating negligible energy transfer between the acceptors. The difference in intensity between A1 and A2 originates from the difference in extinction coefficients ($\varepsilon_{640\ nm}$ = 64000 and 210000 $M^{-1}cm^{-1}$) and quantum yields (= 0.47 and 0.32). (**b**) Histogram of the relative change in fluorescence intensity of A2 after photobleaching of A1 ($\Delta F_{A2} = (\langle N_{A2}\rangle_{before} - \langle N_{A2}\rangle_{after})/\langle N_{A2}\rangle_{after}$, where the subscripts "before" and "after" denote the average intensities before and after photobleaching) for 41 double-labeled ClpB molecules. The red line indicates the mean value of 0.01, demonstrating no inter-acceptor energy transfer.

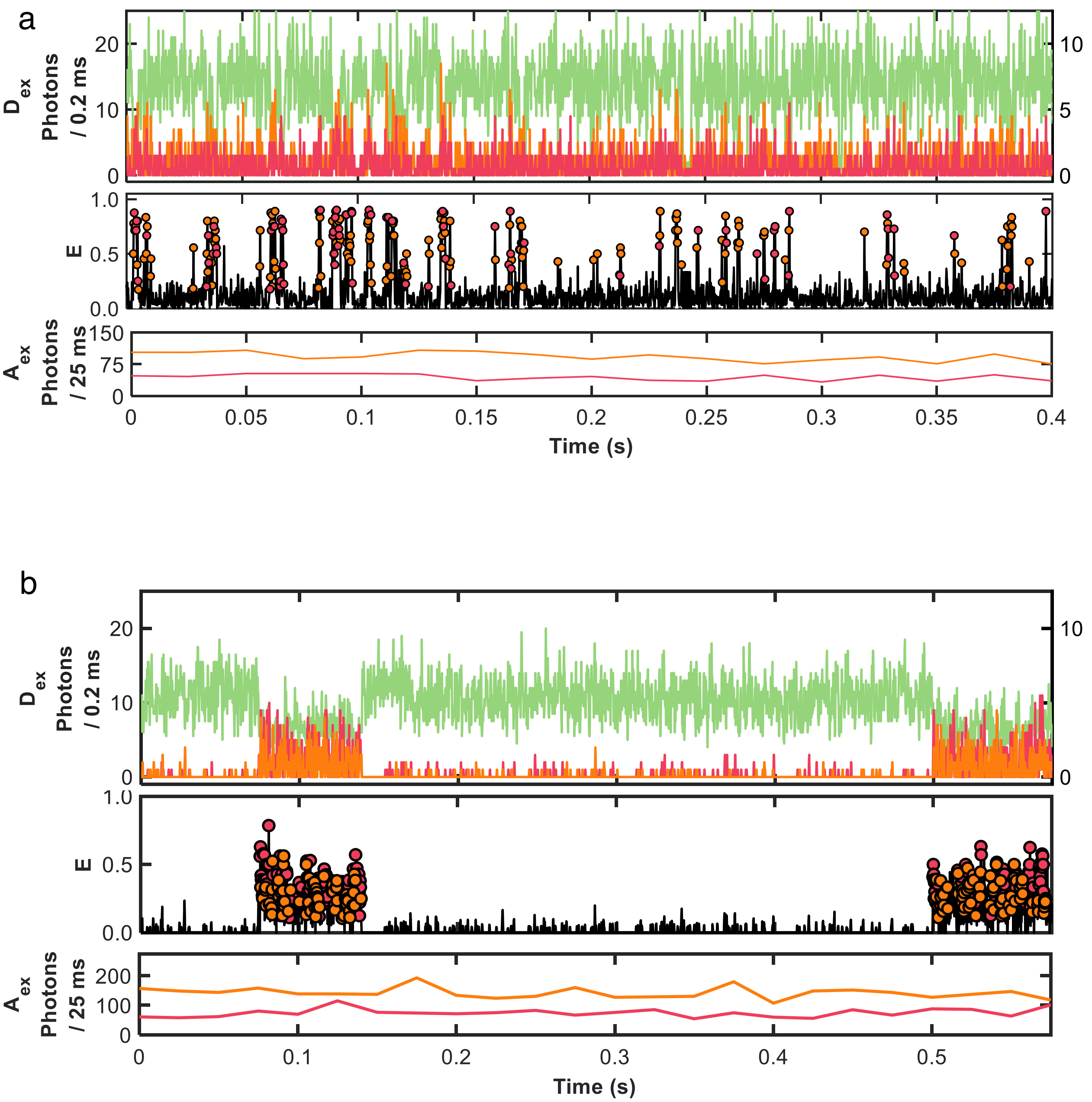

Supplementary Figure 19. Sample trajectories of Cy3B-labeled casein translocating through a double-labeled ClpB. Trajectories are shown in the presence of (**a**) 2 mM ATP and (**b**) 2 mM ATPγS. The fluorescence of the donor (green, left axis) and acceptors (A1 – orange and A2 – red, right axis) after donor excitation (D_{ex}) is shown in the top panel. E_{app} is shown in the middle panel, the colored circles indicate which acceptor is most dominant in each bin during the events. The constant leak-corrected acceptor emission (see Supplementary Figure 18) after acceptor excitation at 640 nm (bottom panels) demonstrates that both acceptors were active throughout the trajectory.

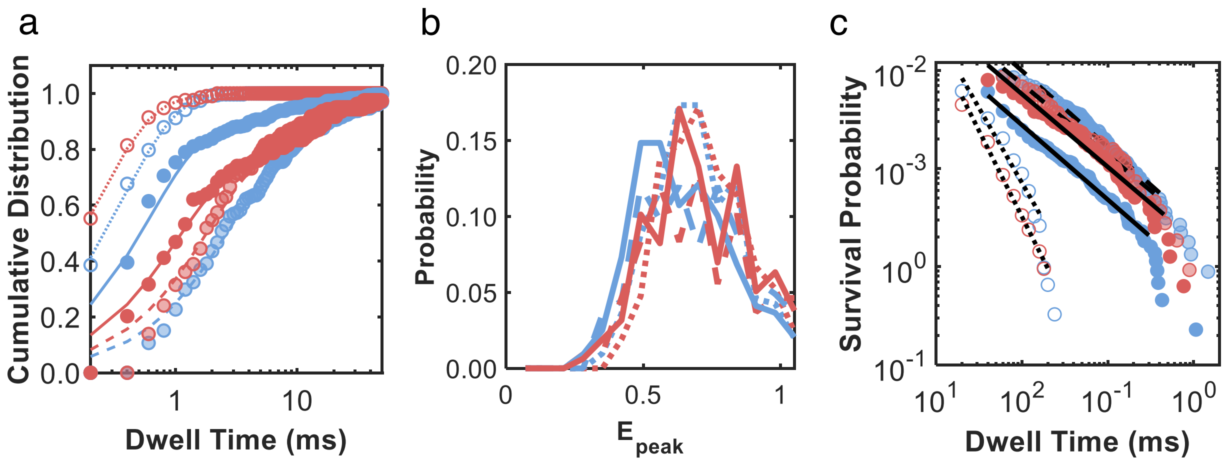

Supplementary Figure 20: Characterization of the different types of interactions in the presence of ATP. Red solid-filled symbols and solid line – type I, blue solid-filled symbols and solid line – type II, red shaded symbols and dashed line – type III, blue shaded symbols and dashed line – type IV, red hollow symbols and dotted line – type V, and blue hollow symbols and dotted line – type VI. (**a**) Cumulative distribution of dwell times, (**b**) histogram of peak FRET efficiencies, and (**c**) power-law distributions. Parameters retrieved from the fits of (**a**) and (**c**) are provided in Supplementary Figure 6.

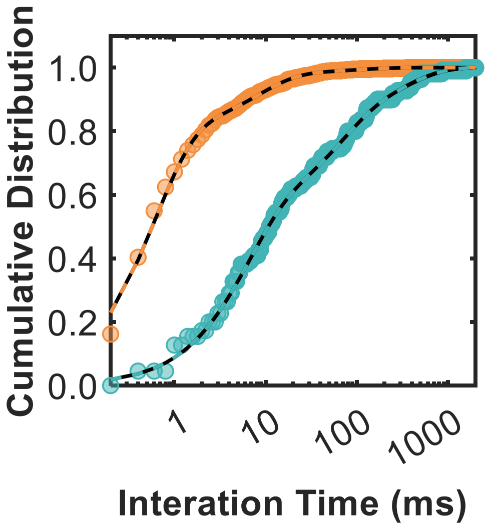

Supplementary Figure 21. Cumulative dwell time histogram of all combined events from the three-color experiments. Cumulative distribution of dwell times in the presence of ATP (orange) and ATPγS (teal). The dashed lines represent the exponential fits corresponding to overall average dwell times of 3.8 and 76 ms with ATP or ATPγS, respectively. Fit parameters are provided in Supplementary Figure 7. Statistics are provided in Supplementary Table 1.

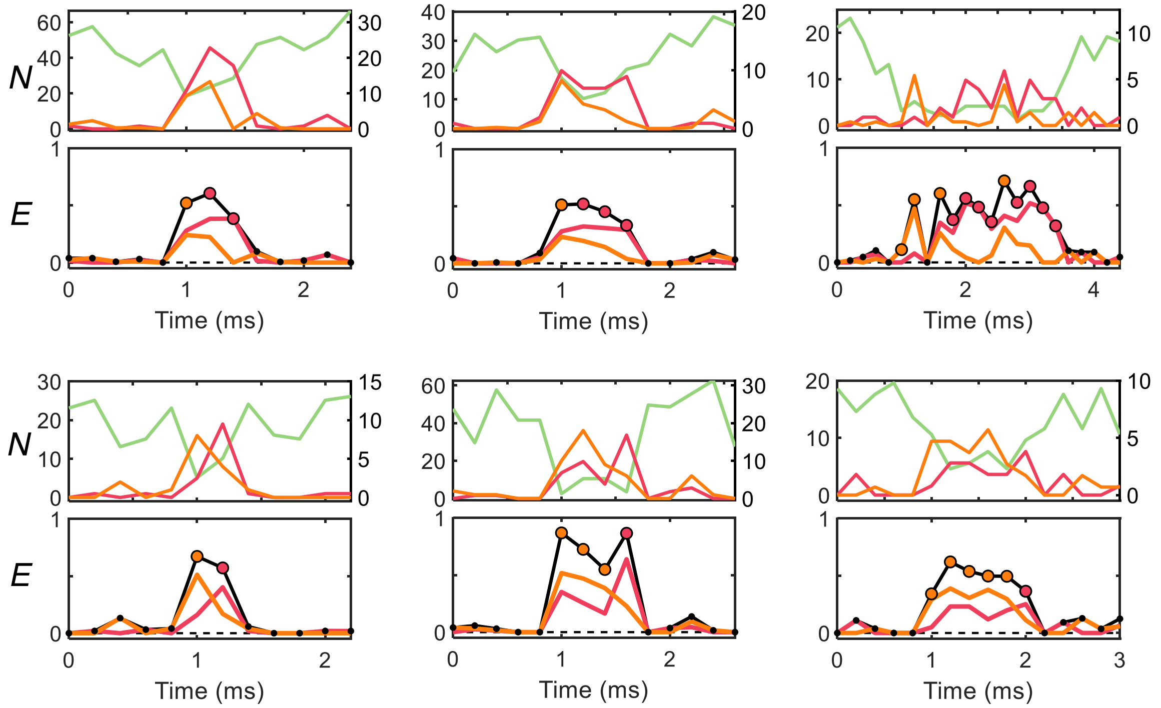

Supplementary Figure 22. Samples of complete forward translocation type I events. The fluorescence of the donor (green, left axis), A1 (orange, right axis), and A2 (red, right axis) are shown in the upper panels with a bin size of 0.2 ms. The lower panels contain the apparent FRET efficiency for A1 (orange), A2 (red), and total (black). The colored circles indicate which acceptor is most dominant in each bin.

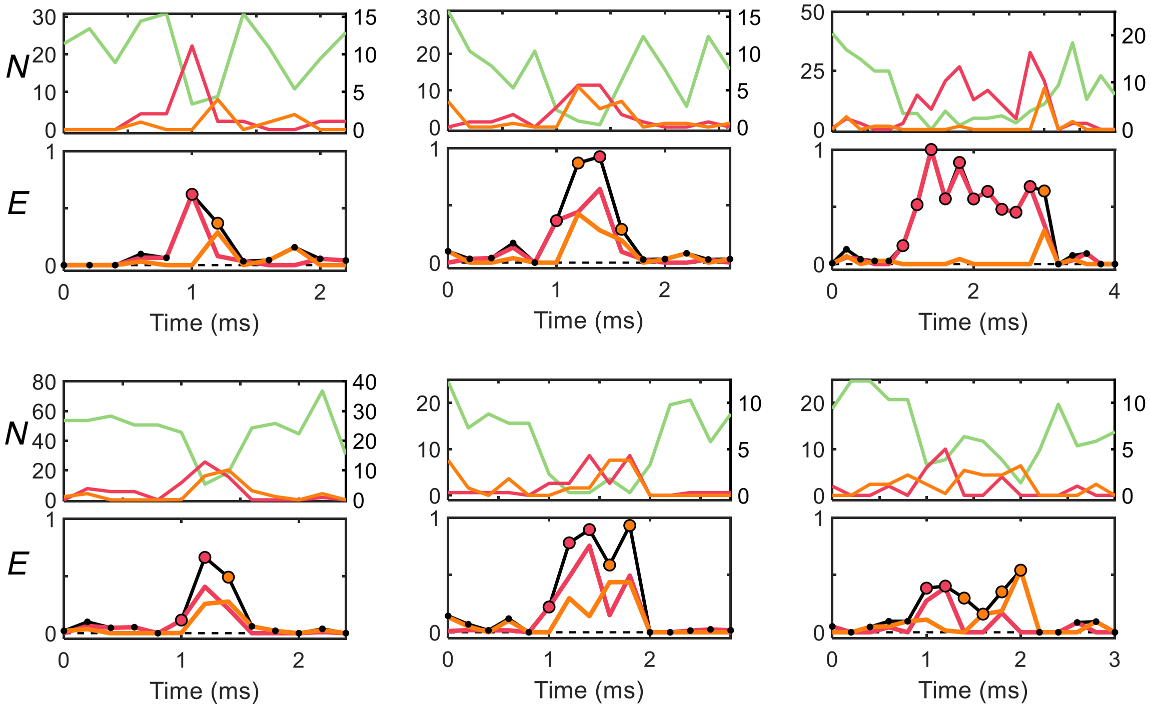

Supplementary Figure 23. Samples of complete reverse translocation type II events. The fluorescence of the donor (green, left axis), A1 (orange, right axis), and A2 (red, right axis) are shown in the upper panels with a bin size of 0.2 ms. The lower panels contain the apparent FRET efficiency for A1 (orange), A2 (red), and total (black). The colored circles indicate which acceptor is most dominant in each bin.

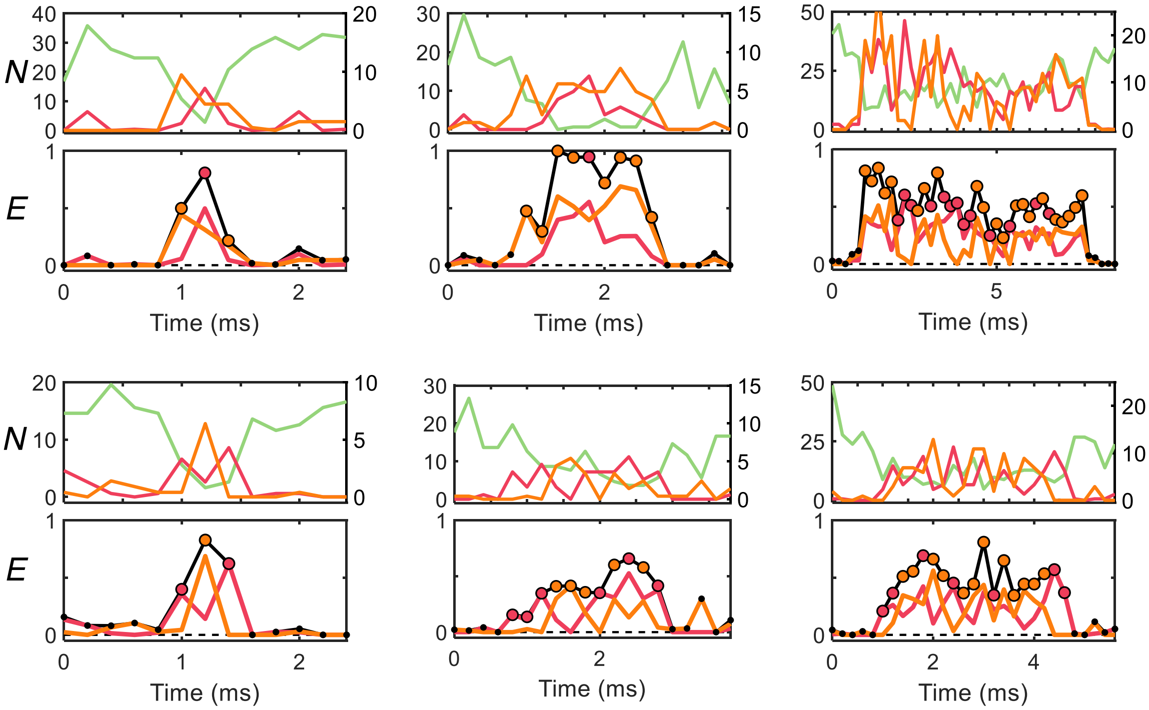

Supplementary Figure 24. Samples of partial translocation types III (top) and IV (bottom) events. The fluorescence of the donor (green, left axis), A1 (orange, right axis), and A2 (red, right axis) are shown in the upper panels with a bin size of 0.2 ms. The lower panels contain the apparent FRET efficiency for A1 (orange), A2 (red), and total (black). The colored circles indicate which acceptor is most dominant in each bin.

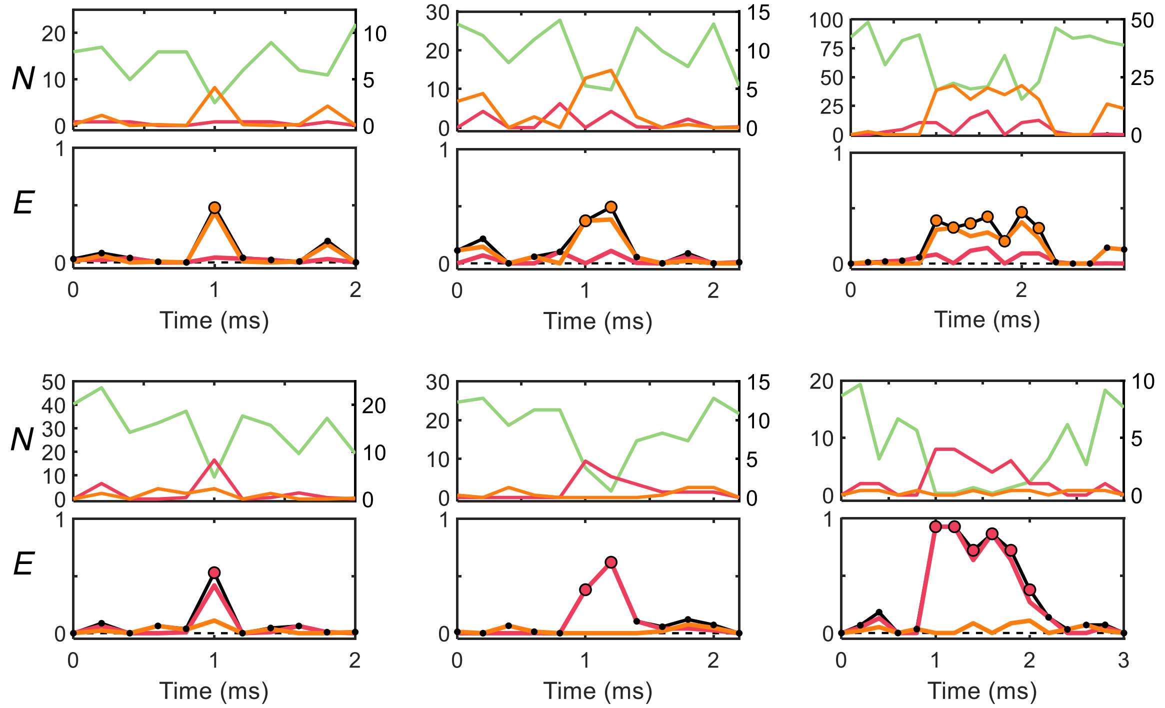

Supplementary Figure 25. Samples of binding types V (top) and VI (bottom) events. The fluorescence of the donor (green, left axis), A1 (orange, right axis), and A2 (red, right axis) are shown in the upper panels with a bin size of 0.2 ms. The lower panels contain the apparent FRET efficiency for A1 (orange), A2 (red), and total (black). The colored circles indicate which acceptor is most dominant in each bin.

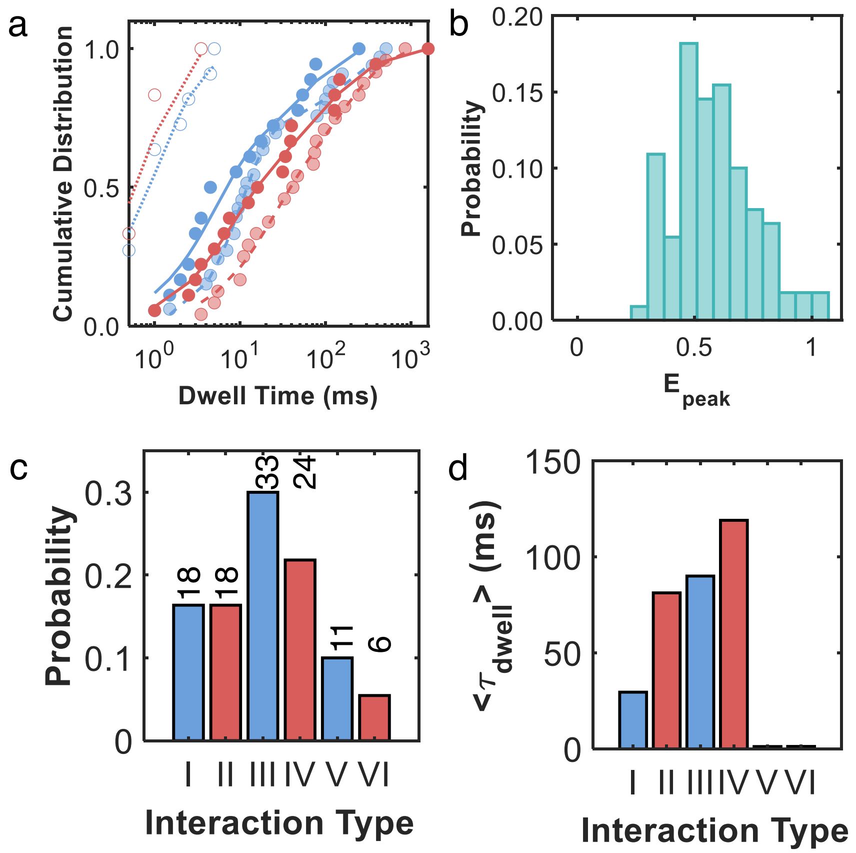

Supplementary Figure 26: Characterization of the different types of events in the three-color experiments in the presence of ATPγS. (**a**) Cumulative distribution of dwell times for type I (red solid-filled symbols and solid line), II (blue solid-filled symbols and solid lines), III (red shaded symbols and dashed lines), IV (blue shaded symbols and dashed lines), V (red hollow symbols and dotted lines), and VI (blue hollow symbols and dotted lines). (**b**) Combined (due to the low number of interactions) peak FRET efficiencies. (**c**) Relative frequency (the numbers above the bars display the number of each type of event) and (**d**) average dwell time of each event type found from the fits in (**a**). Statistics are provided in Supplementary Table 6.

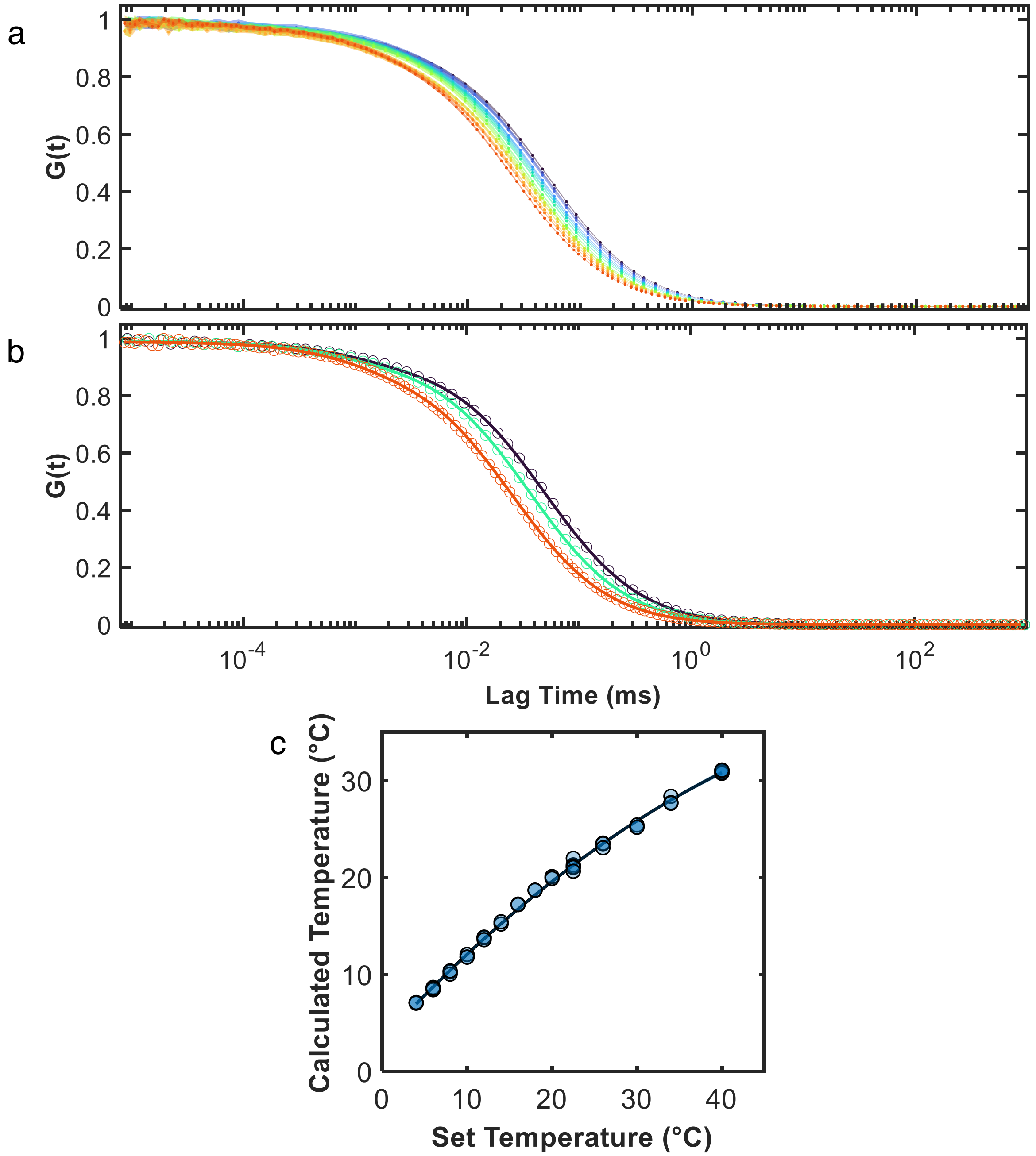

Supplementary Figure 27: Calibration of the temperature cell. (**a**) Fluorescence correlation spectroscopy (FCS) curves of Cy3B in 25 mM HEPES, 25 mM KCl, 10 mM $MgCl_2$ (pH 8). From (dark blue) top to (red) bottom, the temperature cell was set at 4, 6, 8, 10, 12, 14, 16, 20, 22.5, 26, 30, 34, 40 °C. The FCS curves were calculated using the PicoQuant SymPhoTime 64 software. The narrow shaded regions about the curves represent the uncertainty. (**b**) Sample fits (line) of the FCS curves (open circles) with Supplementary Equation (16). To reduce clutter, only temperatures of 4, 16, and 40 °C are shown. (**c**) Calibration curve for the temperature retrieved from Supplementary Equation (17) using the parameters retrieved from the fits of (**a**), corresponding to the temperature inside the confocal volume (Calculated Temperature), as a function of the temperature set on the Peltier controller. Each temperature measurement was repeated in triplicate. Parameters retrieved from the fits of FCS curves are provided in Supplementary Figure 12.

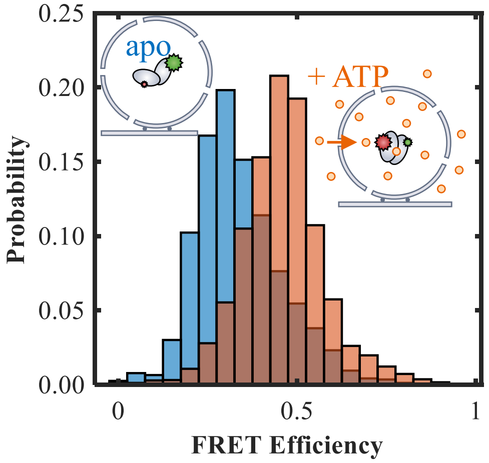

Supplementary Figure 28. SOPC vesicles are permeable to ATP at 10 °C. Histograms of the FRET efficiency values of fluorescently labeled AK variant L82V (3) encapsulated in surface-immobilized 1-stearoyl-2-oleoyl-sn-glycero-3-phosphocholine (SOPC, 18:0-18:1 PC) liposomes at 10 °C without (blue) and with (orange) 2 mM ATP. AK was encapsulated in SOPC liposomes without nucleotides. After immobilization of the liposomes inside a flow cell, the temperature was turned to 10 °C for 15 minutes before data acquisition. Upon the addition of 2 mM ATP, the FRET efficiency histogram of AK, measured initially on 128 individual molecules, shifted to higher values (180 molecules), indicating a population shift that resulted from the SOPC membrane being permeable to ATP at 10 °C. Trajectories were monitored until photobleaching occurred using a circularly polarized 485 nm laser (ca. 200 nW).

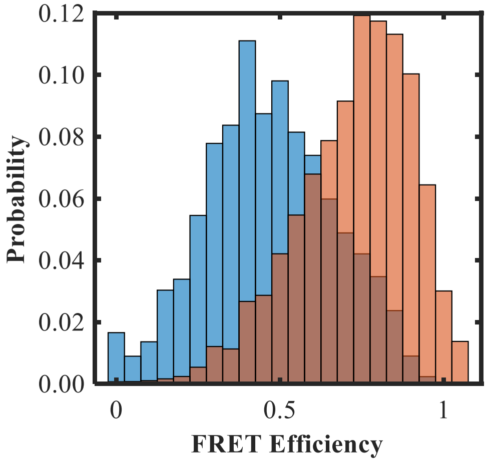

Supplementary Figure 29. 15:0 PC vesicles are permeable to ATP at 32 °C. Histograms of the FRET efficiencies fluorescently labeled AK L82V (3) encapsulated in surface-immobilized (1,2-dipentadecanoyl-sn-glycero-3-phosphocholine) 15:0 PC liposomes at 32 °C without (blue) and with (orange) 2 mM ATP. AK was encapsulated in 15:0 PC liposomes in the absence of ATP. The temperature was set to 32 °C for 20 minutes before data acquisition. Upon the addition of 2 mM ATP, the FRET efficiency histogram of AK, measured initially on 65 molecules, shifted to higher values (157 molecules), indicating that the 15:0 PC membrane was permeable to ATP at 32 °C. Trajectories were monitored until photobleaching occurred using a circularly polarized 485 laser (ca. 200 nW).

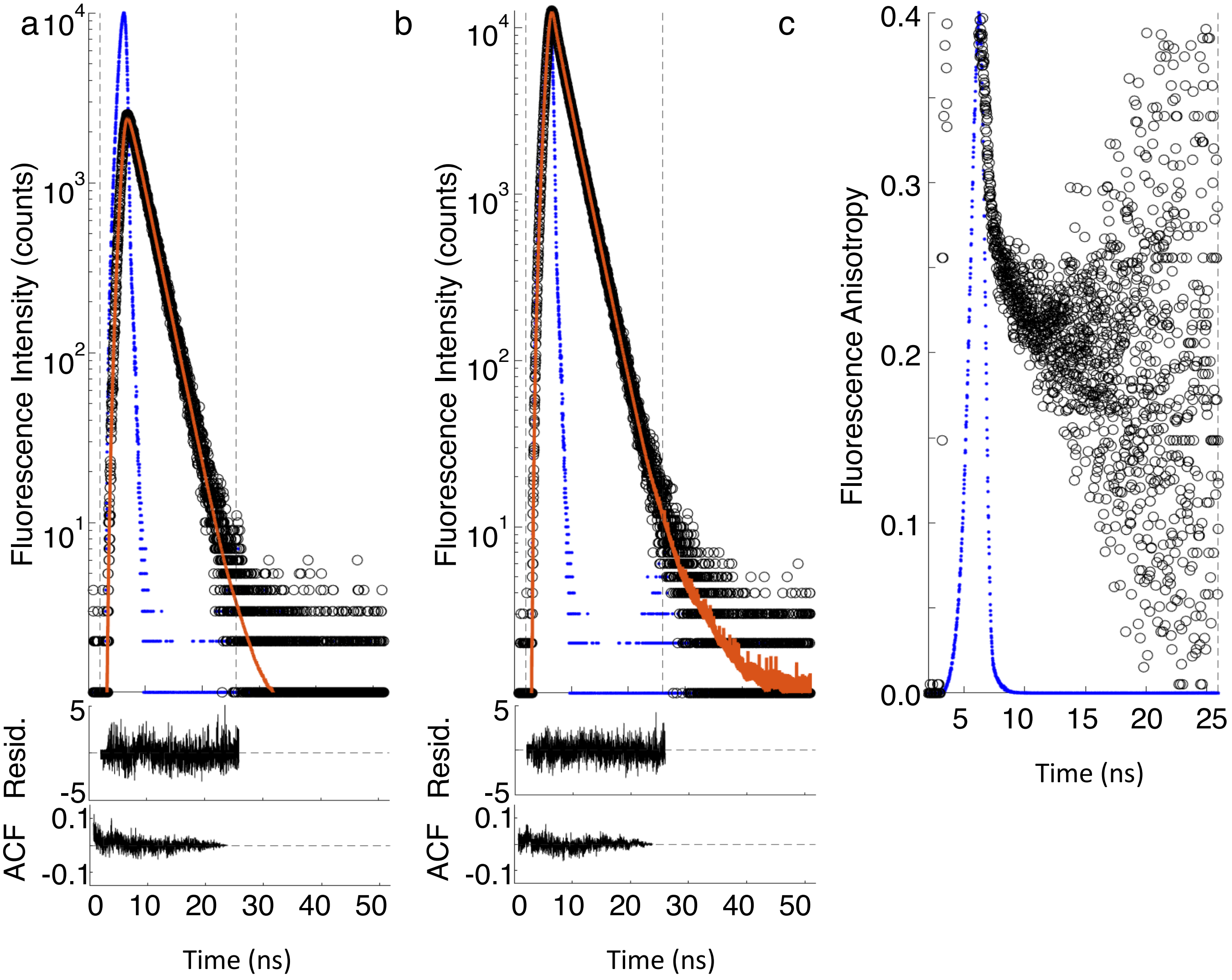

Supplementary Figure 30: Global analysis of the time-resolved fluorescence anisotropy decay curves of ClpB 359. Fluorescence intensity (○) in the (**a**) I_{VH} and (**b**) I_{VV} channels and their corresponding fits to Supplementary Equations (1) and (2) (orange lines) are given alongside (**c**) the fluorescence anisotropy decay. The dashed lines represent data limits used in the global analysis, and the blue dots represent the instrument response function. The normalized residuals (Resid.) and autocorrelation functions (ACF) of the residuals are centered about zero, indicating a good fit. $\chi^2 = 1.07$.

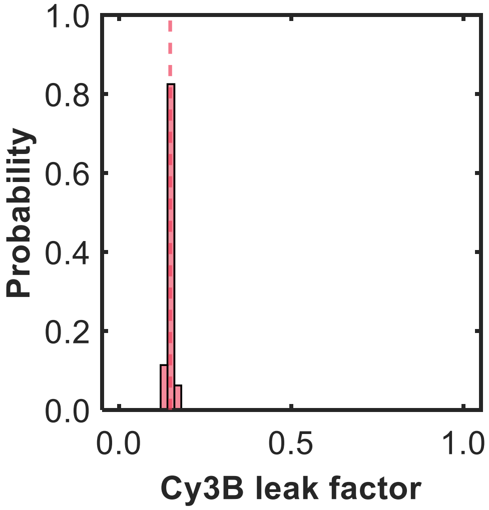

Supplementary Figure 31. The per-molecule Cy3B average leak factor into the acceptor channel in two-color FRET experiments. The histogram is constructed from single-molecule data of 103 Cy3B labeled ClpB molecules, with an average value (dashed line) of 0.148.

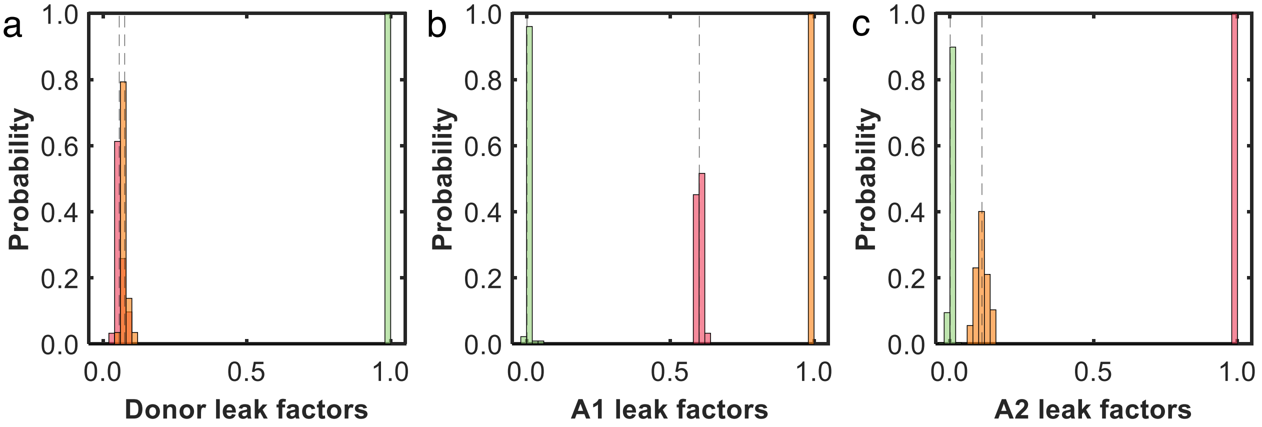

Supplementary Figure 32. The per-molecule average leak factors in the three-color FRET experiments. Single-labeled (**a**) Cy3B (*N* = 154) casein, (**b**) AF647 ClpB (N = 240), and (**c**) CF680R ClpB (*N* = 289) molecules used in the three-color single-molecule experiments. The leak factors were taken as the average values of the distributions. The bar colors indicate the leak of the given species into the donor (Cy3B, green), acceptor 1 (AF647, orange), and acceptor 2 (CF680R, red) channels. The dashed lines represent the average values.

Supplementary Tables

Supplementary Table 1. Summary of the number of labeled ClpB molecules sampled under various experimental conditions.

Dye position	Nucleotide	Nucleotide Concentration (mM)	Temperature (°C)	Number of ClpB molecules	Number of events
NBD1 (359)	ATP	2.0	10	84	1202
			22.5	166	1537
			32	74	532
		0.4	22.5	164	744
		0.2		173	1113
	ATPγS	2.0		147	337
NBD2 (771)	ATP	2.0	10	67	696
			22.5	137	1214
			32	69	621
		0.4	22.5	153	890
		0.2		112	950
	ATPγS	2.0		113	227
NBD1 (176) + NBD2 (771)	ATP	2.0	22.5	135	1445
	ATPγS	2.0		110	135

Supplementary Table 2. Dwell time characterization as a function of temperature and ATP concentration.

Dye position	[ATP] (mM)	Temperature (°C)	Power Law Exponent[a]	Fraction of short events[b]	$\langle\tau_{dwell}\rangle$ (ms)[c]
NBD1 (359)	2.0	10	1.70 ± 0.01	0.88	1.70 ± 0.18
		22.5	1.69 ± 0.02	0.90	1.60 ± 0.13
		32	1.92 ± 0.01	0.93	1.43 ± 0.10
	0.4	22.5	1.46 ± 0.02	0.83	1.87 ± 0.07
	0.2	22.5	1.40 ± 0.01	0.88	1.59 ± 0.08
NBD2 (771)	2.0	10	1.83 ± 0.03	0.89	1.55 ± 0.08
		22.5	1.55 ± 0.02	0.86	1.58 ± 0.14
		32	1.49 ± 0.01	0.88	1.37 ± 0.09
	0.4	22.5	1.42 ± 0.02	0.91	1.94 ± 0.07
	0.2	22.5	1.44 ± 0.02	0.88	1.54 ± 0.09
NBD1 (176) + NBD2 (771)	2.0	22.5	1.68 ± 0.01[d]	0.91	1.32 ± 0.21[d]

[a] Exponents retrieved from the dwell time survival functions.
[b] Fraction of the events which were ≤ 10 ms in length.
[c] Lifetimes retrieved from the exponential fits of the short events (≤ 10 ms).
[d] Overall values. A breakdown of the values for each event type is provided in Supplementary Figure 7.

Supplementary Table 3. Parameters retrieved from the triexponential fits of the combined (NBD1 and NBD2 labeled ClpB) two-color dwell time CDF.

Nucleotide	a_1	τ_1 (ms)	a_2	τ_2 (ms)	a_3	τ_3 (ms)	Weighted Average (ms)
ATP	0.86	1.52	0.11	32.3	0.02	780	21.8
ATPγS	0.30	27.2	0.33	374	0.37	1670	749

Note: The a_i and τ_i values are the preexponential factors and lifetimes for the ith exponent, respectively.

Supplementary Table 4. Parameters retrieved from the triexponential fits of the two-color dwell time CDF as a function of ATP concentration.

[ATP] (mM)	Dye position	a_1	τ_1 (ms)	a_2	τ_2 (ms)	a_3	τ_3 (ms)	Weighted Average (ms)
2.0	NBD1	0.86	1.43	0.12	24	0.02	825	22.1
	NBD2	0.86	1.58	0.13	62	0.01	866	19.6
0.4	NBD1	0.82	1.04	0.12	18	0.06	353	24.2
	NBD2	0.80	1.04	0.10	21	0.10	260	29.1
0.2	NBD1	0.69	1.06	0.17	18	0.14	331	49.1
	NBD2	0.79	1.03	0.11	27	0.10	316	34.7

Note: The a_i and τ_i values are the preexponential factors and lifetimes for the ith exponent, respectively.

Supplementary Table 5. Parameters retrieved from the exponential fits of the frequency of events as a function of ATP concentration.

Dye position	[ATP] (mM)	ATPAse Activity[a] (ATP s^{-1} hexamer^{-1})	Event Frequency[b] (s^{-1})
NBD1 (359)	2.0	1.14 ± 0.03	2.09 ± 0.09
	0.4	0.76 ± 0.05	1.03 ± 0.06
	0.2	0.39 ± 0.03	0.48 ± 0.03
NBD2 (771)	2.0	1.14 ± 0.03	1.63 ± 0.12
	0.4	0.76 ± 0.05	0.74 ± 0.02
	0.2	0.39 ± 0.03	0.49 ± 0.01

[a] With the addition of 3 μM κ-casein.

[b] Average number of events per unit time.

Supplementary Table 6. Summary of parameters characterizing the different types of events.

	2 mM ATP				2 mM ATPγS		
Event Type	Fraction	*N*[a]	τ_{dwell} (ms)[b]	Power Law Exponent[c]	Fraction	*N*[a]	τ_{dwell} (ms)[b]
I	0.30	438	2.1 ± 0.2	1.78	0.17	18	28.0 ± 2.3
II	0.11	158	6.6 ± 1.9	1.73	0.17	18	81.2 ± 16.3
III	0.16	225	11.3 ± 2.1	1.76	0.31	33	90.0 ± 4.4
IV	0.07	108	6.2 ± 0.5	1.74	0.23	24	119.0 ± 17.6
V	0.21	306	0.4 ± 0.1	2.53	0.08	11	1.2 ± 0.1
VI	0.15	210	0.3 ± 0.1	2.75	0.04	6	1.3 ± 0.1

[a] The number of event types observed. Note the limited sampling in the presence of ATPγS due to the long dwell times.
[b] Calculated from the exponential fit of the CDF for the different events.
[c] Exponents retrieved from the dwell time survival functions.

Supplementary Table 7. Parameters retrieved from the triexponential fit of the three-color dwell time CDF in the presence of ATP and ATPγS.

Nucleotide	a_1	τ_1 (ms)	a_2	τ_2 (ms)	a_3	τ_3 (ms)	Weighted Average (ms)
ATP	0.79	0.6	0.19	8.0	0.02	88	3.8
ATPγS	0.53	6.0	0.30	60.5	0.16	332	75.9

Note: The a_i and τ_i values are the preexponential factors and lifetimes for the ith exponent, respectively.

Supplementary Table 8. Translocation velocities estimated from pore loop dynamics.

Pore Loop	PL State 1 Dwell Time[a] (µs)	PL State 2 Dwell Time[a] (µs)	Average PL Cycle Time[b] (ms)	Amplitude of Motion[c] (nm)	Maximum Translocation Speed[d] (*aa* / ms)
PL1	57	68	0.12	1.41	28
PL2	168	101	0.27	1.70	16
PL3	104	88	0.19	1.82	24

[a] All three sets of pore loops (PL) exhibited microsecond dynamics between two different FRET states (16).
[b] The amplitude of motion was estimated using principal component analysis of molecular dynamic simulations (14). The 1 – 2 nm motions agreed well with the change in FRET efficiencies of the two states found in the smFRET experiments (16).
[c] The average time for one up-and-down motion is equal to the sum of the dwell times.
[d] Assumes one *aa* residue corresponds to a contour length of 0.4 nm (17).

Supplementary Table 9. Size distribution of extruded liposomes.

Repeat[a]	z-Average Diameter (nm)	Particle Dispersity Index
1	119	0.04
2	119	0.05
3	118	0.07

[a] Repeats were taken from independently prepared solutions of liposomes.

Supplementary Table 10. Summary of the steady-state fluorescence anisotropy of fluorescently labeled ClpB.

Species	Steady-State Anisotropy	$r_{SS,calc}$[a]
Cy3B free dye	0.097 ± 0.01	-
ClpB 359CyB	0.271 ± 0.01	0.26
ClpB 771Cy3B	0.277 ± 0.01	0.30
ClpB 771CF680R	0.227 ± 0.02	-
Casein CF660R	0.133 ± 0.03	-

[a] Calculated steady-state anisotropy value (Supplementary Equation (5)) using parameters retrieved from the time-resolved fluorescence analysis (Supplementary Figure 11).

Supplementary Table 11. Parameters retrieved from the global analysis of the fluorescence anisotropy decays of Cy3B-labeled ClpB.

Parameter	ClpB 359 Cy3B	ClpB 771Cy3B
χ^2	1.07	1.11
τ_D (ns)	2.7	2.8
G	0.51	
φ_F (ns)†	0.95	1.11
contribution (α) of the fast tumbling time	0.45	0.31
φ_P (ns)††	210	
$r_{SS,calc}$[a]	0.26	0.30

† Fast tumbling time associated with rotational freedom of the dye.
†† The slow tumbling time of ClpB occurs on a time scale much slower than the decay of Cy3B. A tumbling time of 210 ns, predicted by the Perrin equation for a 12 nm diameter sphere, was used as a fixed value in the analysis (18).
[a] Calculated steady-state fluorescence anisotropy values (Supplementary Equation (5)).

Supplementary Table 12: Parameters retrieved from the FCS curves of Cy3B at different temperatures. A description of the model (Equations S15 and S16) and parameters is provided above in the SI text under *Fluorescence correlation spectroscopy*).

Set T (°C)[a]	ω	f_T	τ_T (µs)	N	τ_D (µs)	$G_\infty \times 10^4$	Cell T (°C)[b]
4	6.3	0.064 ± 0.001	1.0 ± 0.1	1.07 ± 0.01	49.8 ± 0.1	-2 ± 2	7.08 ± 0.04
6		0.061 ± 0.001	1.0 ± 0.2	1.07 ± 0.05	47.4 ± 0.2	-1 ± 2	8.6 ± 0.1
8		0.061 ± 0.003	1.0 ± 0.1	1.07 ± 0.04	44.9 ± 0.3	-3 ± 1	10.2 ± 0.2
10		0.062 ± 0.002	1.0 ± 0.2	1.07 ± 0.05	42.7 ± 0.2	-1 ± 2	11.9 ± 0.2
12		0.060 ± 0.001	1.1 ± 0.2	1.07 ± 0.02	40.3 ± 0.2	-2 ± 2	13.7 ± 0.1
14		0.060 ± 0.001	1.0 ± 0.1	1.07 ± 0.03	38.5 ± 0.2	-2 ± 1	15.3 ± 0.2
16		0.059 ± 0.001	0.8 ± 0.1	1.07 ± 0.03	36.4 ± 0.1	-1 ± 1	17.22 ± 0.04
18		0.058 ± 0.001	0.9 ± 0.1	1.06 ± 0.01	34.9 ± 0.1	-2 ± 1	18.68 ± 0.02
20		0.054 ± 0.002	0.8 ± 0.1	1.07 ± 0.02	33.6 ± 0.1	0 ± 1	20.0 ± 0.1
22.5		0.071 ± 0.014	0.8 ± 0.2	1.09 ± 0.21	32.2 ± 0.3	-1 ± 2	21.2 ± 0.5
26		0.081 ± 0.001	0.8 ± 0.1	1.10 ± 0.04	30.1 ± 0.2	0 ± 1	23.4 ± 0.3
30		0.078 ± 0.002	0.8 ± 0.1	1.10 ± 0.04	28.6 ± 0.1	1 ± 2	25.3 ± 0.1
34		0.076 ± 0.001	1.0 ± 0.2	1.09 ± 0.03	26.7 ± 0.3	1 ± 1	27.9 ± 0.4
40		0.077 ± 0.001	1.1 ± 0.2	1.10 ± 0.05	24.7 ± 0.1	2 ± 1	32.0 ± 0.2

[a] Temperature set on the Peltier control unit.

[b] Temperature calculated (Supplementary Equation 17) inside the confocal volume.

Supplementary Table 13. ATPase activity of ClpB in the presence of 2 mM ATP.

Protein	ATPase Activity (ATP min^{-1} monomer^{-1})	
	– κ-casein	+ 3 µM κ-casein
WT	3.50 ± 0.37	11.4 ± 0.3
359C	2.59 ± 0.19	12.8 ± 0.4
771C	1.56 ± 0.08	7.5 ± 0.2
176C + 771C	3.45 ± 0.09	10.1 ± 0.3
359Cy3B	2.36 ± 0.08	7.3 ± 0.4
771Cy3B	1.00 ± 0.10	4.8 ± 0.1
176AF647 + 771CF680R	1.18 ± 0.03	6.8 ± 0.3

Supplementary Table 14. Parameters retrieved from the fit of the ATPase activity by the Hill equation.

Conditions	K_m (µM)	n
– κ-casein	0.42 ± 0.01	2.74 ± 0.11
+ 3 µM κ-casein	0.31 ± 0.02	1.87 ± 0.17

Supplementary References

1. E. Boukobza, A. Sonnenfeld, G. Haran, Immobilization in surface-tethered lipid vesicles as a new tool for single biomolecule spectroscopy. *Journal of Physical Chemistry B* **105**, 12165-12170 (2001).
2. P.-A. Monnard, D. W. Deamer, Nutrient Uptake by Protocells: A Liposome Model System. *Origins of life and evolution of the biosphere* **31**, 147-155 (2001).
3. D. Scheerer *et al.*, Allosteric communication between ligand binding domains modulates substrate inhibition in adenylate kinase. *Proceedings of the National Academy of Sciences* **120** (2023).
4. H. Mazal *et al.*, Tunable microsecond dynamics of an allosteric switch regulate the activity of a AAA+ disaggregation machine. *Nature Communications* **10**, 1438 (2019).
5. J. Yoo, J. M. Louis, I. V. Gopich, H. S. Chung, Three-Color Single-Molecule FRET and Fluorescence Lifetime Analysis of Fast Protein Folding. *The Journal of Physical Chemistry B* **122**, 11702-11720 (2018).
6. J. F. Nagle, S. Tristram-Nagle, Structure of lipid bilayers. *Biochimica et Biophysica Acta (BBA) - Reviews on Biomembranes* **1469**, 159-195 (2000).
7. H. Little *et al.*, Simplification in the Acquisition and Analysis of Fluorescence Decays Acquired with Polarized Emission for Time-Resolved Fluorescence Anisotropy Measurements. *Analytical Chemistry* **92**, 668-673 (2020).
8. J. R. Lakowicz, *Principles of Fluorescence Spectroscopy* (2006), 10.1007/978-0-387-46312-4.
9. J. J. Moré, D. C. Sorensen, Computing a Trust Region Step. *SIAM Journal on Scientific and Statistical Computing* **4**, 553-572 (1983).
10. B. Gilboa *et al.*, Confinement-Free Wide-Field Ratiometric Tracking of Single Fluorescent Molecules. *Biophysical Journal* **117**, 2141-2153 (2019).
11. A. A. Choi, L. Xiang, W. Li, K. Xu, Single-Molecule Displacement Mapping Indicates Unhindered Intracellular Diffusion of Small (less, similar1 kDa) Solutes. *J Am Chem Soc* 10.1021/jacs.3c00597 (2023).
12. T. K. G. Dabir S. Viswanath , Dasika H. L. Prasad , Nidamarty V.K. Dutt , Kalipatnapu Y. Rani, *Viscosity of Liquids* (Springer Netherlands, 2007), 10.1007/978-1-4020-5482-2.
13. O. Krichevsky, G. Bonnet, Fluorescence correlation spectroscopy: the technique and its applications. *Reports on Progress in Physics* **65**, 251-297 (2002).
14. M. Iljina *et al.*, Single-molecule FRET probes allosteric effects on protein-translocating pore loops of a AAA+ machine. *Biophysical Journal* **123**, 374-388 (2024).
15. S. Schlee, Y. Groemping, P. Herde, R. Seidel, J. Reinstein, The chaperone function of ClpB from Thermus thermophilus depends on allosteric interactions of its two ATP-binding sites11Edited by A. R. Fersht. *Journal of Molecular Biology* **306**, 889-899 (2001).
16. H. Mazal, M. Iljina, I. Riven, G. Haran, Ultrafast pore-loop dynamics in a AAA+ machine point to a Brownian-ratchet mechanism for protein translocation. *Science Advances* **7**, eabg4674 (2021).
17. S. R. K. Ainavarapu *et al.*, Contour Length and Refolding Rate of a Small Protein Controlled by Engineered Disulfide Bonds. *Biophysical Journal* **92**, 225-233 (2007).
18. J. R. Lakowicz, "Time-Dependent Anisotropy Decays" in Principles of Fluorescence Spectroscopy, J. R. Lakowicz, Ed. (Springer US, Boston, MA, 2006), 10.1007/978-0-387-46312-4_11 chap. Chapter 11, pp. 383-412.
